# Pretreatment AMH–AFC stratification of poor-ovarian-response risk in young women with diminished ovarian reserve: a multicentre retrospective cohort study

**DOI:** 10.3389/fendo.2026.1968836

**Published:** 2026-09-28

**Authors:** Yanning Lv, Jiahao Fang, Haiyan Yang, Jianying He, Lihua Xia, Honglian Wu

**Affiliations:** 1 Department of Reproductive Medicine, The First Affiliated Hospital of Lishui University, Lishui People's Hospital, Lishui, Zhejiang, China; 2 Department of Reproductive Medicine, The First Affiliated Hospital of Wenzhou Medical University, Wenzhou, Zhejiang,, China; 3 Department of Reproductive Medicine, Jinhua People's Hospital, Jinhua, Zhejiang, China; 4 Department of Reproductive Medicine, Huzhou Maternal and Child Health Hospital, Huzhou, Zhejiang, China

**Keywords:** anti-Müllerian hormone, antral follicle count, diminished ovarian reserve, nomogram, poor ovarian response, risk stratification

## Abstract

In women aged 35 years or younger with diminished ovarian reserve (DOR), we evaluated whether two pretreatment markers, anti-Müllerian hormone (AMH) and antral follicle count (AFC), can stratify the risk of poor ovarian response (POR) before stimulation. This retrospective cohort included IVF/ICSI cycles from four centres in Zhejiang, China (2022–2025). Of 1,754 DOR-eligible cycles, 157 were removed in two *post-hoc* phenotype-cleaning exclusions (PCOS, n = 139; AMH ≥5 ng/mL without PCOS, n = 18), leaving 1,597 cycles. POR was defined as ≤3 oocytes. Cycles were cross-classified by AFC ≤5 and AMH <0.5 ng/mL, and a two-marker logistic model was fitted. POR occurred in 42.0% of cycles. Total AFC (adjusted OR 0.843) and ln (AMH) (OR 0.310) carried the main pretreatment signal; age and basal FSH added little. The two single-marker cells had similar observed POR rates (46.4% vs 49.6%; P = 0.56), but equivalence was not established. The AFC ≤5 boundary was reproduced in the seed-2024 70:30 split and was re-selected in 92.0% of 1,000 bootstrap samples when the cut-off was chosen in-bag and evaluated out-of-bag. The descriptive three-tier rates were 23.9%, 47.1%, and 78.8%; when all 1,754 eligible cycles were retained, the middle-tier rate fell to 39.5%. For the fixed two-marker specification, bootstrap optimism correction gave an AUC of 0.768; centre-held-out AUCs using verified record-level centre labels ranged from 0.725 to 0.800. AMH and AFC therefore provide moderate pretreatment risk stratification within this young DOR cohort, but the absolute tier risks remain sensitive to cohort definition and cut-off choice. External validation and recalibration are required before clinical use.

## 1 Introduction

Lifetime infertility prevalence is estimated at approximately 17.5% globally (1). In assisted reproductive technology, cumulative live-birth potential rises with the number of embryos and oocytes generated during treatment, with oocyte yield showing a strong association with live birth (2–4). Knowing before treatment how a woman is likely to respond is therefore central to counselling and to planning.

Diminished ovarian reserve (DOR) is characterised by a reduced follicular pool, reflected in a low antral follicle count (AFC) and low anti-Müllerian hormone (AMH) (5, 6). It is traditionally associated with advancing age, yet it is increasingly recognised in women under 35 (7, 8). Compared with age-matched women with normal reserve, young women with DOR retrieve fewer oocytes and have lower live-birth rates (8); their oocytes also show reduced euploidy independently of age (9). Age remains an important determinant of prognosis among poor responders generally (10), but its range is narrow in a cohort already restricted to women aged 35 years or younger, so its incremental discrimination may be limited. The Bologna criteria (11) and the POSEIDON classification (12) standardised how low-prognosis patients are identified; POSEIDON Group 3 captures young women with low prestimulation ovarian-reserve biomarkers (12, 13). AMH and AFC are the principal reserve markers used to predict ovarian response (14, 15), yet the amount of absolute POR-risk information carried by their concordant and discordant patterns within young DOR has had little dedicated study.

Several prediction models for poor ovarian response or retrieval failure already exist. Xu et al. developed a model for unexpected poor response that combined baseline reserve markers with treatment-choice variables, including stimulation protocol and initial gonadotrophin dose (16). Ye et al. studied failed oocyte retrieval in POSEIDON Groups 3 and 4 using AMH, protocol and intracycle variables such as trigger-day follicle count and the oestradiol change during stimulation (17). These are useful models, but they answer a later question in the treatment pathway. A study particularly close to ours is Teng et al. (18), which used AMH and AFC cut-offs to identify a ‘high-risk DOR’ group among women aged ≤35 years whose reserve was mildly reduced but had not yet reached the POSEIDON DOR range; its focus was on recognising that earlier reserve phenotype and on subsequent reproductive outcomes. Our cohort begins one step later: every included cycle already meets our AMH/AFC definition of DOR, and we ask how much pretreatment information AMH and AFC provide about POR (≤3 oocytes), especially when the two markers disagree, before a stimulation regimen has been selected. Discordance is common and the two markers are not necessarily interchangeable; AFC has been reported to predict oocyte yield more strongly when they disagree (19). The contribution here is therefore not a new DOR definition. It is an estimate of POR risk across concordant and discordant AMH/AFC patterns within established young DOR, together with an assessment of how far a simple two-marker model can stratify that risk using information available at the first consultation.

## 2 Materials and methods

### 2.1 Design, participants, and outcomes

We conducted a retrospective cohort study of women aged ≤35 years undergoing IVF/ICSI at four reproductive medicine centres in Zhejiang Province, China, between January 2022 and December 2025. Each centre de-identified its records locally before transfer to the lead centre. The restored analytic file retains both a de-identified participant_id and a record-level centre code (Lishui, Wenzhou, Jinhua or Huzhou), so centre-specific analyses no longer depend on participant-number blocks. The pooled registry contained 10,084 stimulation-cycle records, all with a recorded AFC. Among the 1,754 DOR-eligible records, all 1,754 participant IDs were distinct; the 1,597-cycle primary cohort likewise contained 1,597 distinct IDs. The contributing centres reported retaining only the first eligible cycle per woman within the study window. The restored IDs support a one-record-per-ID structure, although independently de-identified IDs cannot completely rule out the same woman being represented under different IDs across centres. The stimulation cycle is therefore retained as the unit of analysis.

For this study, DOR was operationally defined as AMH <1.2 ng/mL or total AFC <7. The individual AMH <1.2 ng/mL and AFC <7 cut-points have precedent in DOR literature (8), but their use here in a logical OR rule is study-specific rather than a universal DOR definition. We excluded records with BMI <18.5 or >30 kg/m^2^ (n = 1,274), zero or missing AFC (n = 69), no DOR by the AMH/AFC definition (n = 6,970) and missing menstrual-cycle length (n = 17), leaving 1,754 DOR-eligible cycles from 1,754 distinct participant IDs. The BMI range was an eligibility criterion applied at the first screening step, before the DOR definition (**Figure 1**; **Supplementary Note S1.4**). Two additional phenotype-cleaning exclusions were then made during *post-hoc* data cleaning, after outcome data were available: PCOS (Rotterdam criteria; n = 139) and AMH ≥5 ng/mL without PCOS (n = 18). These exclusions were based on pretreatment characteristics, but they were not outcome-blind or prospectively registered. Together they removed 157 of 1,754 records (9.0%), leaving 1,597 cycles (**Figure 1**). We therefore repeated the analysis in the full 1,754-cycle cohort without either exclusion (**Supplementary Table S9**). Further detail on these decisions and record provenance is given in **Supplementary Note S1**.

**Figure 1:**
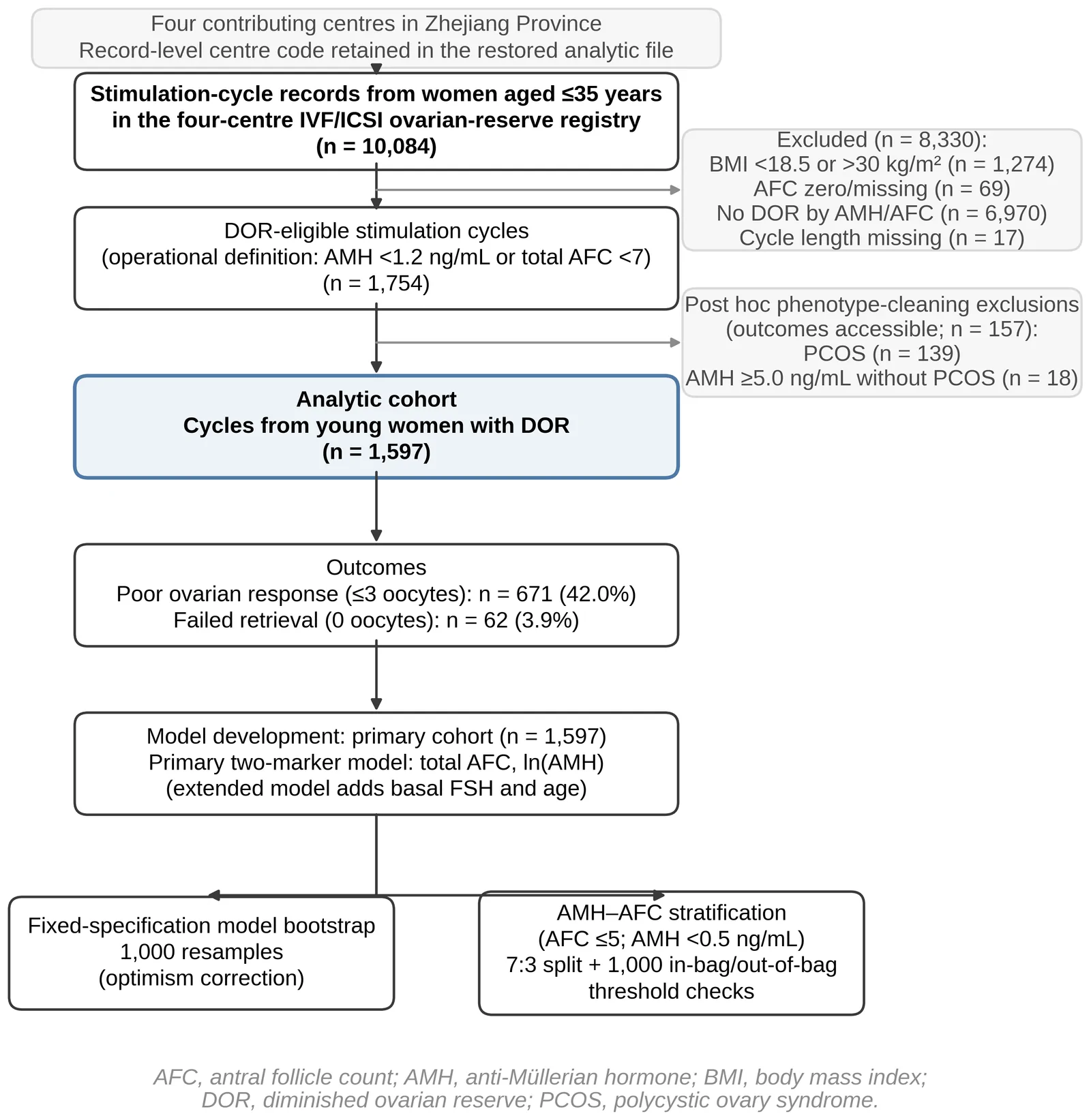
Screening, eligibility, and analytic cohort. The registry pooled 10,084 cycles from four centres. Of these, 1,274 were excluded for BMI outside 18.5–30 kg/m^2^, 69 for zero or missing AFC, 6,970 for not meeting the AMH/AFC definition of DOR (AMH <1.2 ng/mL or total AFC <7) and 17 for missing menstrual-cycle length, leaving 1,754 eligible cycles. Two additional exclusions were then made during *post hoc* phenotype cleaning after outcomes were accessible: PCOS (n = 139) and AMH ≥5 ng/mL without PCOS (n = 18). The analytic cohort comprised 1,597 cycles, of which 671 (42.0%) had POR (≤3 oocytes) and 62 (3.9%) had failed retrieval (0 oocytes). Record-level centre codes were available in the restored analytic file and were used for the revised centre analyses. The bootstrap branch depicts optimism correction for the fixed model specification; threshold stability was assessed separately by split-sample and in-bag/out-of-bag analyses. AFC, antral follicle count; AMH, anti-Müllerian hormone; BMI, body mass index; DOR, diminished ovarian reserve; ICSI, intracytoplasmic sperm injection; IVF, *in vitro* fertilisation; PCOS, polycystic ovary syndrome; POR, poor ovarian response.

The primary outcome was poor ovarian response, defined as ≤3 oocytes retrieved, following the Bologna and POSEIDON criteria (11, 12). The secondary outcome was failed retrieval, operationally defined as 0 oocytes, consistent with previous failed-oocyte-retrieval literature (17). Terminology for ART procedures otherwise followed the International Glossary on Infertility and Fertility Care (20). Because failed retrieval was rare, it was analysed exploratorily rather than treated as a confirmatory endpoint. The stimulation cycle is the unit of analysis throughout, and results are reported in terms of cycles.

The lead ethics approval for this retrospective secondary analysis was issued on 16 June 2026, after the 2022–2025 clinical records had been generated in routine care but before study-specific extraction, pooling and analysis of those records. The study was conducted in accordance with the Declaration of Helsinki, and the requirement for informed consent was waived (see Ethics statement).

### 2.2 Variables

All values were those recorded at the first consultation. Serum AMH was measured in ng/mL on the routine clinical platform of the contributing centre. Platforms were not harmonised. The de-identified dataset does not carry a per-cycle record of which platform was used, and no cross-platform calibration or split-sample comparison between centres was available to us, so between-centre comparability of AMH cannot be assessed here and the AMH thresholds reported are assay-dependent. Hormones were assayed on cycle days 2–4, and AFC was the total number of 2 mm to 10 mm follicles in both ovaries, counted on transvaginal ultrasound on cycle days 2–4 by the attending sonographer.

Candidate predictors were restricted to variables available before stimulation: age, BMI, infertility duration, total AFC, AMH, basal FSH, basal LH, basal oestradiol, FSH/LH ratio and menstrual-cycle length. All ten were examined in univariable analyses (**Supplementary Table S1**) and then entered into the LASSO; a GnRH-antagonist-protocol indicator was added as an eleventh LASSO candidate. The univariable analyses were descriptive and were not used to remove predictors before LASSO. Left and right AFC appear in **Table 1** but were never offered to a model, since they sum exactly to total AFC. Protocol was not included in the pretreatment nomogram because it is selected after the reserve assessment and may itself lie downstream of AMH/AFC in the clinical decision pathway. It was examined only in a sensitivity-adjusted model.

**Table 1:** Baseline characteristics of the primary analytic cohort, by ovarian response.

Characteristic	Overall (n = 1,597)	Normal response (>3 oocytes, n = 926)	Poor response (≤3 oocytes, n = 671)	P
Age (years), mean ± SD	32.1 ± 2.7	32.0 ± 2.7	32.3 ± 2.7	0.016
Infertility duration (years), median (IQR)	3 (2–4)	3 (1–4)	3 (2–4)	0.256
BMI (kg/m^2^), mean ± SD	22.9 ± 2.8	22.7 ± 2.8	23.1 ± 2.8	0.004
Left AFC (n), mean ± SD	3.2 ± 2.0	3.7 ± 2.1	2.7 ± 1.6	<0.001
Right AFC (n), mean ± SD	3.4 ± 2.3	4.0 ± 2.4	2.7 ± 2.0	<0.001
Total AFC (n), median (IQR)	6 (4–8)	6 (5–10)	5 (3–6)	<0.001
AMH (ng/mL), median (IQR)	0.78 (0.49–1.05)	0.93 (0.67–1.12)	0.54 (0.31–0.83)	<0.001
Basal FSH (IU/L), median (IQR)	8.07 (6.35–10.72)	7.70 (6.23–10.05)	8.54 (6.81–11.75)	<0.001
Basal E2 (pmol/L), median (IQR)	121 (70–196)	120 (71–186)	123 (70–211)	0.204
Basal LH (IU/L), median (IQR)	4.17 (2.94–5.92)	4.10 (2.91–5.79)	4.29 (3.02–6.13)	0.100
FSH/LH ratio, median (IQR)	2.09 (1.44–2.89)	2.00 (1.41–2.78)	2.19 (1.54–3.07)	0.001
Menstrual cycle length (days), median (IQR)	30 (28–30)	30 (28–30)	30 (28–30)	0.001
Oocytes retrieved (n), median (IQR)	4 (2–7)	6 (5–8)	2 (1–3)	<0.001
GnRH-antagonist protocol, n (%)	460 (28.8)	329 (35.5)	131 (19.5)	<0.001
Tubal factor infertility, n (%)	735 (46.0)	459 (49.6)	276 (41.1)	0.001
Endometriosis, n (%)	125 (7.8)	86 (9.3)	39 (5.8)	0.014

Continuous variables are reported as mean ± SD or median (interquartile range); categorical variables as n (%). Comparisons are between cycles with a normal response (>3 oocytes) and those with a poor response (≤3 oocytes), using the independent-samples t-test, Mann–Whitney U test or χ^2^ test as appropriate. P-values are two-sided. Menstrual-cycle length has identical medians and interquartile ranges in the two groups; the nominal P value reflects the sample size, not a clinically meaningful difference. AFC, antral follicle count; AMH, anti-Müllerian hormone; BMI, body mass index; E2, oestradiol; FSH, follicle-stimulating hormone; GnRH, gonadotrophin-releasing hormone; IQR, interquartile range; LH, luteinising hormone; SD, standard deviation.

### 2.3 Statistical analysis

Continuous variables are reported as mean ± SD or median (IQR), categorical variables as n (%). Groups were compared with the independent-samples t-test, Mann–Whitney U test or χ^2^ test as appropriate, with P <0.05 (two-sided) taken as significant. Missing data were handled by complete-case analysis. AMH was strongly right-skewed (skewness 2.53) and was modelled on the natural-log scale (skewness of ln[AMH] −1.08); a linear term would force a constant odds ratio per ng/mL across the whole range, which is untenable when the clinically important variation lies between approximately 0.1 ng/mL and 1 ng/mL.

Univariable logistic regression and single-predictor AUCs were used to describe candidate associations and discrimination; they were not used as a filtering rule. LASSO regression with 5-fold cross-validation then included all ten pretreatment candidates plus the GnRH-antagonist-protocol indicator to inspect the relative stability of signals among correlated variables (**Supplementary Figure S1**). AMH and AFC had the largest standardised coefficients, while the remaining terms were substantially smaller or zero. Following this data-informed review, four first-consultation predictors—total AFC, ln(AMH), basal FSH and age—were evaluated in an extended model. Because FSH and age added little incremental value (Results), the parsimonious total AFC + ln(AMH) specification became the primary model. This simplification was not fully pre-specified. No further P-value-based deletion was performed within the displayed two-marker fit; the four-variable model is reported separately in **Supplementary Tables S2**, **S4**.

The fixed primary two-marker specification was fitted on the primary analytic cohort and assessed by bootstrap resampling (1,000 replicates), with optimism correction of the AUC and calibration slope. The extended four-variable specification was assessed in the same way, and a stratified 7:3 split-sample analysis (seed 2024) is reported as a sensitivity analysis (**Supplementary Tables S4**, **S6**). In both model bootstraps the displayed specification was refitted in each resample; the preceding LASSO and model-simplification steps were not repeated. The corrected performance estimates therefore quantify optimism conditional on the chosen specification rather than uncertainty from the entire model-development process (21, 22). Discrimination was assessed by the AUC, calibration by a flexible calibration curve, decile-based calibration, calibration slope and intercept, the Brier score, and the Hosmer–Lemeshow test, and clinical utility by decision curve analysis. AUCs were compared with DeLong’s test.

For combined stratification, women were cross-classified by total AFC (≤5 vs >5) and AMH (<0.5 vs ≥0.5 ng/mL). The AFC cut-off was informed by POSEIDON’s published AFC <5 definition (12), but our use of ≤5 is a study-specific operational boundary rather than the identical POSEIDON criterion. AMH <0.5 ng/mL was chosen on clinical grounds to represent the severe end of the DOR range. Because AFC ≤5 also equals the Youden-optimal value in the primary analytic cohort, we added two analyses that separate cut-off selection from evaluation. First, in the seed-2024 stratified 70:30 split, the AFC cut-off was selected in the training set and then applied unchanged to the held-out validation set; AFC ≤5 was optimal in both partitions (**Supplementary Table S6**). Second, in 1,000 bootstrap repetitions, the AFC cut-off was re-selected in each in-bag sample and the resulting tier rule was evaluated only in the corresponding out-of-bag observations. AFC ≤5 was selected in 92.0% of repetitions (≤4 in 8.0%); mean out-of-bag POR rates across repetitions were 24.4%, 47.3%, and 79.2% for the three tiers. These analyses reduce, but do not remove, uncertainty about the exact boundary because they remain internal. Sensitivity analyses also used AMH thresholds of 0.78 ng/mL (the cohort Youden value) and 1.0 ng/mL (**Supplementary Table S5**). Rates were compared by χ^2^, with the two single-marker cells assessed in a 1-degree-of-freedom contrast. A non-significant difference is not evidence of equivalence, so we also used exploratory two one-sided tests (TOST) with 10- and 15-percentage-point margins. These margins were not pre-specified and are used only to show how strongly any equivalence claim depends on the tolerance chosen. Trend across ordered AFC strata was tested by Cochran–Armitage, and the rare failed-retrieval outcome by Firth penalised-likelihood regression with two predictors.

Centre-level analyses used the record-level centre code in the restored file. We compared POR rates across centres, added centre indicators to the primary two-marker model, and performed internal–external cross-validation by leaving one centre out in turn and refitting the same fixed two-marker specification in the remaining three centres (**Supplementary Table S8**; **Supplementary Figure S3**). Because predictor selection and model simplification were not repeated within each held-out-centre cycle, these results assess the transportability of the fixed specification rather than the entire development pathway (21, 22). Other *post hoc* functional-form checks were carried out within the extended four-variable model: restricted cubic-spline tests for each continuous predictor and a total AFC × ln(AMH) interaction (**Supplementary Table S10**), followed by a linear-versus-spline AFC comparison that retained ln(AMH), basal FSH and age (**Supplementary Table S11**). These analyses assess the functional-form robustness of the extended model and are not a direct spline refit of the primary two-marker calculator. The study had no registered analysis plan. The candidate set and AMH threshold were based on clinical rationale; the exact AFC ≤5 operational boundary was informed by, but is not identical to, POSEIDON’s <5 criterion. The two phenotype-cleaning exclusions were *post-hoc* and were made while outcome data were accessible. Their effect on the tier estimates is quantified in the *Results* and **Supplementary Table S9**.

Analyses used Python 3.12 (NumPy 2.4, pandas 3.0, SciPy 1.17, scikit-learn 1.8, Matplotlib 3.10) with a fixed seed of 2024. During revision, we used OpenAI ChatGPT (model/version GPT-5.6 Sol; source: OpenAI) and Anthropic Claude (model/version Claude Opus 5; source: Anthropic) for language editing, manuscript restructuring and consistency checks. ChatGPT was also used to assist with the execution and checking of author-specified re-analyses of the de-identified dataset, including threshold resampling and centre-level validation. The authors defined the statistical procedures, checked the generated code and outputs against the source data, and retained responsibility for every analytic and interpretive decision. Scripts and source-data files needed to reproduce the reported results are supplied as **Supplementary Material**. Reporting follows TRIPOD and STROBE (21, 23); both checklists are supplied as **Supplementary Material**.

## 3 Results

### 3.1 Cohort

Of 10,084 stimulation-cycle records from women aged ≤35 years in the registry, 1,754 met the study’s AMH/AFC definition of DOR, and 1,597 remained after the two *post hoc* phenotype-cleaning exclusions (**Figure 1**). Poor ovarian response occurred in 671 cycles (42.0%) and failed retrieval in 62 (3.9%). Median AMH was 0.78 ng/mL (IQR 0.49–1.05), and median total AFC was 6 (IQR 4–8).

Poor responders had lower AFC and AMH, a higher basal FSH and FSH/LH ratio, marginally higher age and BMI, and were less often given a GnRH-antagonist protocol (**Table 1**). Menstrual-cycle length had the same median and interquartile range in both groups (30 days [28–30]); its nominal P value is not accompanied by a clinically meaningful separation of the distributions.

### 3.2 Predictors and the multivariable model

On univariable analysis, ln(AMH) and total AFC discriminated better than the other pretreatment candidates (AUC 0.730 and 0.701; **Supplementary Table S1**, **Supplementary Figure S2**). In the cross-validated LASSO, these two variables also had the largest standardised coefficients; the remaining candidate coefficients were much smaller or zero (**Supplementary Figure S1**).

In the primary two-marker model (**Table 2**), total AFC (adjusted OR 0.843 per follicle, 95% CI 0.811–0.876) and ln(AMH) (adjusted OR 0.310 per unit, 95% CI 0.258–0.373) were each associated with lower odds of POR (both P <0.001). Adding basal FSH and age gave little incremental value: basal FSH had an adjusted OR of 1.008 (P = 0.55), age had an adjusted OR of 1.043 (P = 0.052), and their joint contribution was not significant (likelihood-ratio χ^2^ = 4.3, df = 2, P = 0.12; **Supplementary Tables S2**, **S10**). Additional adjustment for GnRH-antagonist protocol left the reserve-marker estimates similar (0.853 and 0.323), while the protocol itself was associated with lower odds of POR (adjusted OR 0.617, 95% CI 0.475–0.800, P <0.001; **Supplementary Table S7**). This protocol coefficient is not interpretable as a treatment effect because protocol choice was non-random and may reflect both confounding by indication and downstream treatment selection.

**Table 2:** Primary two-marker multivariable logistic regression model for poor ovarian response (development cohort, n = 1,597).

Predictor	Adjusted OR (95% CI)	P
Total AFC (per 1 follicle)	0.843 (0.811–0.876)	<0.001
ln(AMH) (per 1-unit increase)	0.310 (0.258–0.373)	<0.001

Primary two-marker model fitted on the primary analytic cohort (1,597 cycles, 671 POR events). AMH is entered as the natural logarithm. Model equation: logit(P) = 0.305 − 0.171 × AFC − 1.170 × ln(AMH), with AFC in follicles and AMH in ng/mL. Performance for this fixed specification: apparent AUC 0.769 (95% CI 0.745–0.793); bootstrap optimism-corrected AUC 0.768 (1,000 replicates); calibration slope 1.00 (corrected 1.00); calibration intercept 0.00; Brier score 0.191; Hosmer–Lemeshow χ^2^ = 5.68, df = 8, P = 0.68. The two-marker specification was selected after comparison with the four-variable model and is therefore data-informed. The bootstrap refitted the fixed two-marker specification but did not repeat the preceding LASSO/model-simplification step; its optimism correction is conditional on this final specification. No further P-value-based deletion was performed within this fit. With 671 events and two final coefficients, the nominal EPV is 335.5, but this ratio does not account for uncertainty from the preceding model-selection process. Basal FSH and age were evaluated in the extended model and added little incremental value (likelihood-ratio χ^2^ = 4.3, df = 2, P = 0.12); their coefficients and validation are in **Supplementary Tables S2**, **S4**. In a sensitivity model additionally adjusted for GnRH-antagonist protocol (adjusted OR 0.617, 95% CI 0.475–0.800, P <0.001), the AFC and ln(AMH) estimates were 0.853 and 0.323 (**Supplementary Table S7**). AFC, antral follicle count; AMH, anti-Müllerian hormone; AUC, area under the curve; CI, confidence interval; EPV, events per variable; OR, odds ratio; POR, poor ovarian response.

### 3.3 AMH–AFC stratification

Poor-response rates fell monotonically across AFC strata, from 74.9% at AFC 1–3 to 18.2% at AFC ≥11 (Cochran–Armitage χ^2^ for trend = 188.31, df = 1, P <0.001; **Figure 2A**).

**Figure 2:**
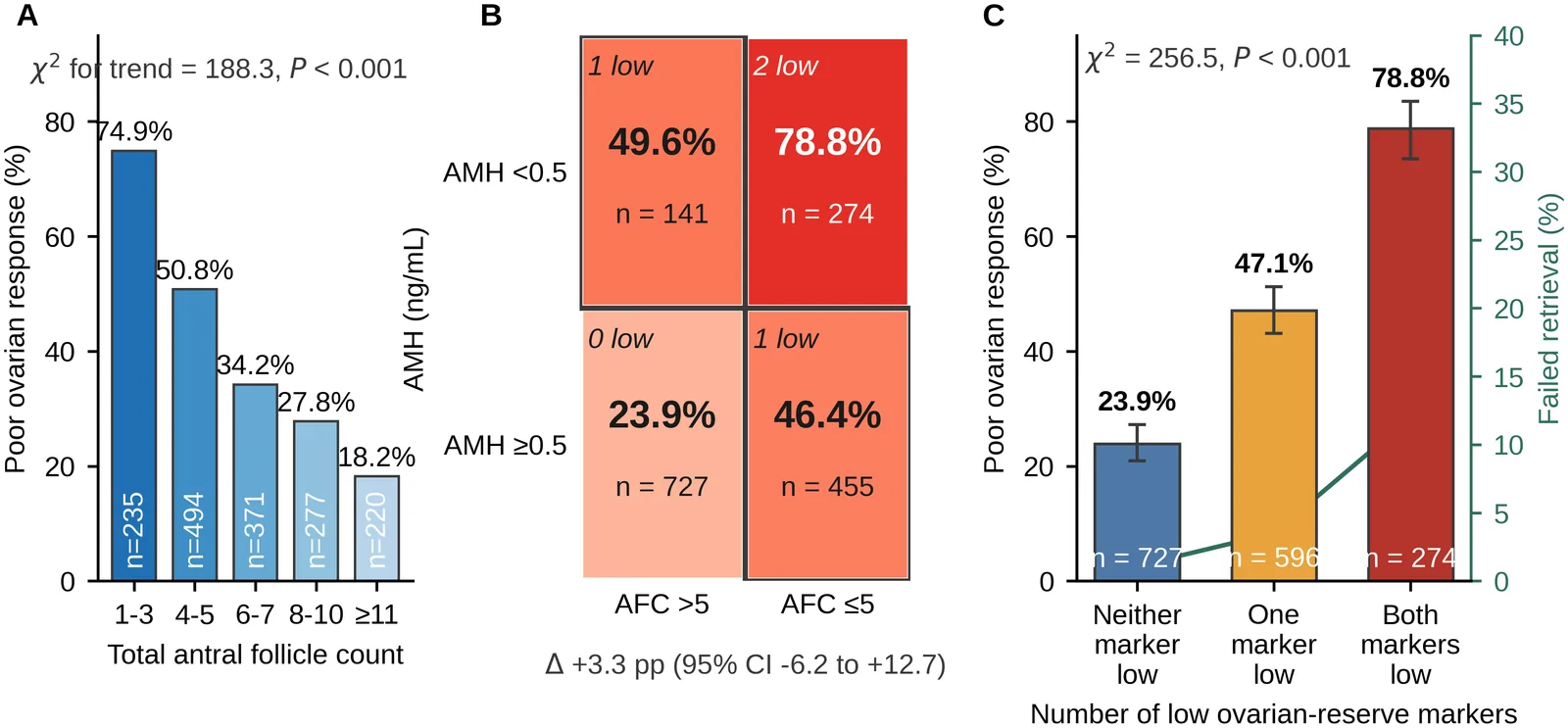
AMH–AFC stratification of poor ovarian response. **(A)** POR rate across AFC strata (Cochran–Armitage χ^2^ for trend = 188.31, df = 1, P <0.001). **(B)** Cross-classification by AFC ≤5 and AMH <0.5 ng/mL; colour encodes the POR rate and each cell gives the rate and group size. The two single-marker cells (outlined) had similar observed POR rates (46.4% vs 49.6%; absolute difference 3.3 percentage points, 95% CI −6.2 to +12.7; χ^2^ = 0.34, df = 1, P = 0.56), but equivalence was not established. **(C)** Descriptive three-tier summary obtained by merging those two cells. Bars show POR rates with 95% confidence intervals, and the line on the right axis shows failed-retrieval rates (χ^2^ = 256.45, df = 2, P <0.001 for POR across tiers). Median oocyte yield is reported in **Table 3** and is not represented by the right-axis line. The merge is threshold-dependent; the four-cell results are retained in **Table 3**. AFC, antral follicle count; AMH, anti-Müllerian hormone; POR, poor ovarian response.

Cross-classifying by AFC ≤5 and AMH <0.5 ng/mL gave four cells with clearly different POR rates overall (χ^2^ = 256.92, df = 3, P <0.001; **Table 3**, **Figure 2B**). The two single-marker cells had similar observed rates: 46.4% (211/455) when AFC alone was low and 49.6% (70/141) when AMH alone was low, an absolute difference of 3.3 percentage points (95% CI −6.2 to +12.7; χ^2^ = 0.34, df = 1, P = 0.56), with a median yield of four oocytes in both. The confidence interval remains wide because the AMH-low-only cell contains 141 cycles. Exploratory TOST was compatible with equivalence within a 15-percentage-point margin (P = 0.007) but not within 10 points (P = 0.081); these margins were not pre-specified. We therefore treat the similarity as an observed, threshold-specific finding rather than proof that the two discordant states are equivalent. The four cells remain reported separately in **Table 3**.

**Table 3:** Cross-classification by AFC and AMH, and the resulting three-tier scheme.

Group	n (%)	Poor ovarian response, n (%)	Failed retrieval, n (%)	Oocytes, median (IQR)
Cross-classification (four cells)				
AFC >5 and AMH ≥0.5	727 (45.5)	174 (23.9)	8 (1.1)	6 (4–8)
AFC ≤5 and AMH ≥0.5 (AFC low only)	455 (28.5)	211 (46.4)	18 (4.0)	4 (2–6)
AFC >5 and AMH <0.5 (AMH low only)	141 (8.8)	70 (49.6)	3 (2.1)	4 (2–5)
AFC ≤5 and AMH <0.5	274 (17.2)	216 (78.8)	33 (12.0)	2 (1–3)
Three-tier scheme (single-marker cells merged)				
Tier 1 — neither marker low	727 (45.5)	174 (23.9; 20.9–27.2)	8 (1.1)	6 (4–8)
Tier 2 — one marker low	596 (37.3)	281 (47.1; 43.1–51.2)	21 (3.5)	4 (2–6)
Tier 3 — both markers low	274 (17.2)	216 (78.8; 73.5–83.5)	33 (12.0)	2 (1–3)

Thresholds: total AFC ≤5; AMH <0.5 ng/mL. Poor ovarian response, ≤3 oocytes; failed retrieval, 0 oocytes. Percentages in the POR column are within-group rates, with 95% confidence intervals shown for the three tiers. Across the four cells, χ^2^ = 256.92, df = 3, P <0.001. The two single-marker cells had similar observed rates (46.4% vs 49.6%; absolute difference 3.3 percentage points, 95% CI −6.2 to +12.7; χ^2^ = 0.34, df = 1, P = 0.56) and the same median oocyte yield (4 in both). Exploratory TOST was compatible with equivalence within a 15-percentage-point margin (P = 0.007) but not within 10 points (P = 0.081); the margins were not pre-specified and equivalence is therefore not established, and the four cells are reported separately. The three-tier merge is a descriptive, threshold-specific summary. Across the three tiers, χ^2^ = 256.45, df = 2, P <0.001. In the full 1,754-cycle sensitivity cohort, without the two *post hoc* phenotype-cleaning exclusions, the corresponding tier rates are 23.8%, 39.5% and 79.0% (**Supplementary Table S9**). AFC, antral follicle count; AMH, anti-Müllerian hormone; IQR, interquartile range; POR, poor ovarian response.

For descriptive stratification, merging the two single-marker cells yields three tiers (χ^2^ = 256.45, df = 2, P <0.001; **Table 3**, **Figure 2C**). Observed POR rates were 23.9% (95% CI 20.9–27.2) with neither marker low, 47.1% (43.1–51.2) with one low and 78.8% (73.5–83.5) with both low; median yields were six, four, and two oocytes. Failed retrieval followed the same ordering, from 1.1% to 3.5% to 12.0%.

Sensitivity analyses show that the three-tier summary depends on both threshold choice and cohort definition. Raising the AMH threshold to 0.78 or 1.0 ng/mL preserved the broad risk gradient, but at 0.78 ng/mL the two single-marker cells differed (33.2% vs 41.4%, P = 0.048), so the rationale for merging them no longer held (**Supplementary Table S5**). The AFC boundary itself was more stable: in the seed-2024 70:30 split, AFC ≤5 was Youden-optimal in both the training and held-out validation sets. Applying the training-derived cut-off with AMH <0.5 ng/mL to the validation set gave tier rates of 20.6%, 55.0%, and 78.2%. In the 1,000-replicate bootstrap in-bag/out-of-bag threshold analysis, AFC ≤5 was re-selected in 92.0% of repetitions, and the mean out-of-bag tier rates remained ordered at 24.4%, 47.3%, and 79.2%. Retaining all 1,754 eligible cycles nevertheless changed the middle-tier rate from 47.1% to 39.5%, while the outer-tier rates were 23.8% and 79.0% (**Table 3** **footnote**; **Supplementary Table S9**). Refitting the same primary two-marker model in those 1,754 cycles gave an AFC OR of 0.846 (95% CI 0.814–0.879), an ln(AMH) OR of 0.275 (0.232–0.326), and an apparent AUC of 0.795. The marker associations therefore remain similar, but the absolute middle-tier risk is materially dependent on cohort definition, and the single-marker merge remains dependent on the AMH cut-off.

### 3.4 Model performance, clinical utility, and failed retrieval

The primary two-marker model is presented as a nomogram in **Figure 3**. Apparent discrimination was an AUC of 0.769 (95% CI 0.745–0.793); for this fixed two-marker specification, bootstrap optimism correction gave an AUC of 0.768 and a corrected calibration slope of 1.00. The Brier score was 0.191, the Hosmer–Lemeshow test showed no evidence of gross miscalibration (χ^2^ = 5.68, df = 8, P = 0.68), and the internal calibration curve tracked the diagonal reasonably closely (**Figure 4**). The model discriminated better than either marker alone: 0.769 vs 0.730 for ln(AMH) and 0.701 for total AFC. DeLong differences were +0.039 (95% CI 0.024 to 0.054) and +0.068 (0.047 to 0.088), both P <0.001 (**Figure 5A**). Decision curve analysis showed greater net benefit than treat-all or treat-none across thresholds around the observed event rate (**Figure 5B**), but this does not demonstrate that using the model to make treatment decisions improves outcomes. All performance estimates are internal to the development cohort, and the bootstrap correction does not include uncertainty from the preceding data-informed simplification.

**Figure 3:**
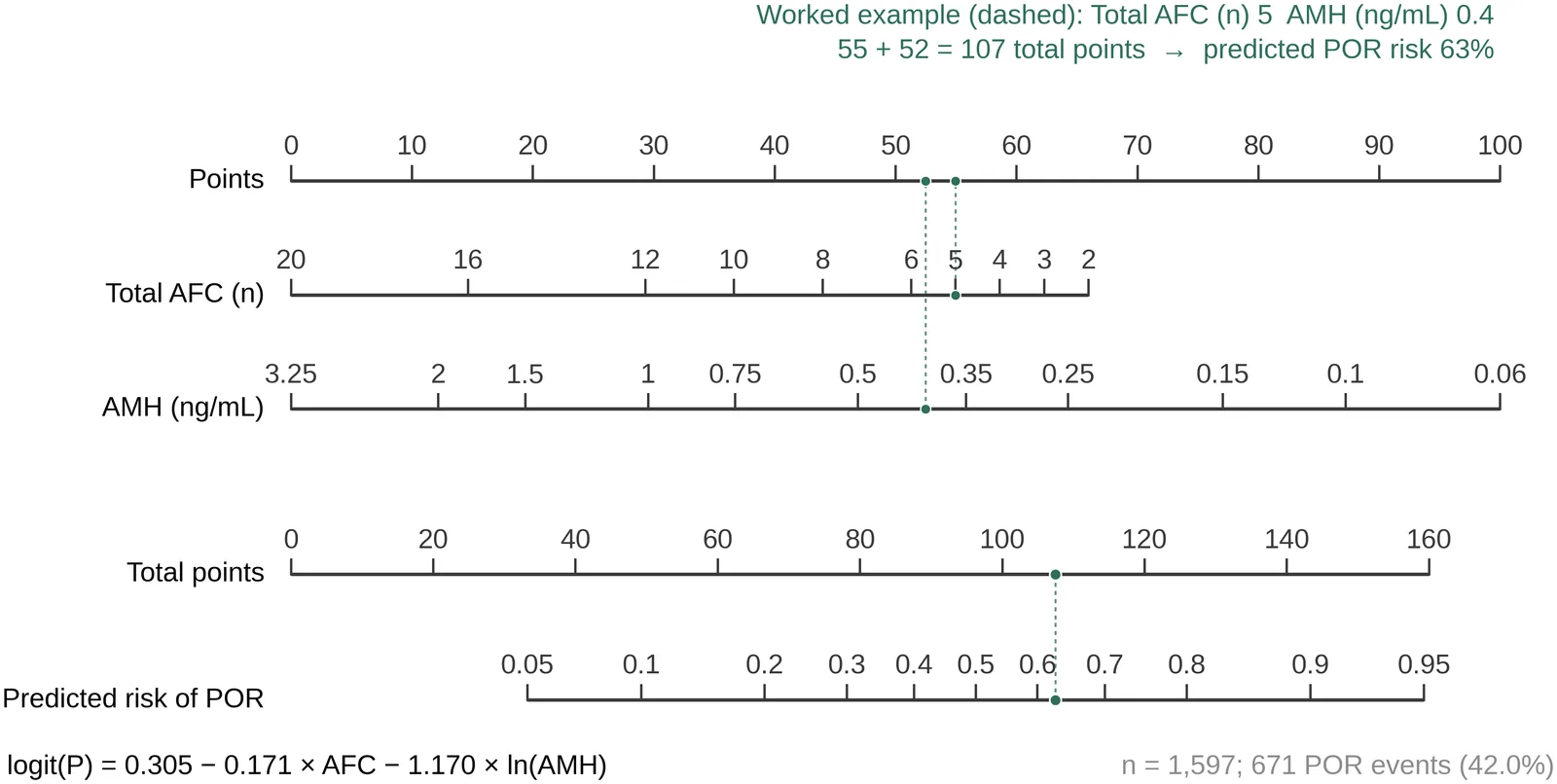
Pretreatment nomogram for the primary two-marker model (poor ovarian response, ≤3 oocytes). Points are read from the total AFC and AMH axes up to the Points ruler, summed, and located on the Total points axis to give the predicted risk. The AMH axis is non-linear because AMH enters the model as ln(AMH). The dashed path shows a worked example (AFC 5, AMH 0.40 ng/mL → 107 points → 63% risk). Model equation: logit(P) = 0.305 − 0.171 × AFC − 1.170 × ln(AMH), with AFC in follicles and AMH in ng/mL. The extended four-variable model is shown in **Supplementary Figure S4**. AFC, antral follicle count; AMH, anti-Müllerian hormone.

**Figure 4:**
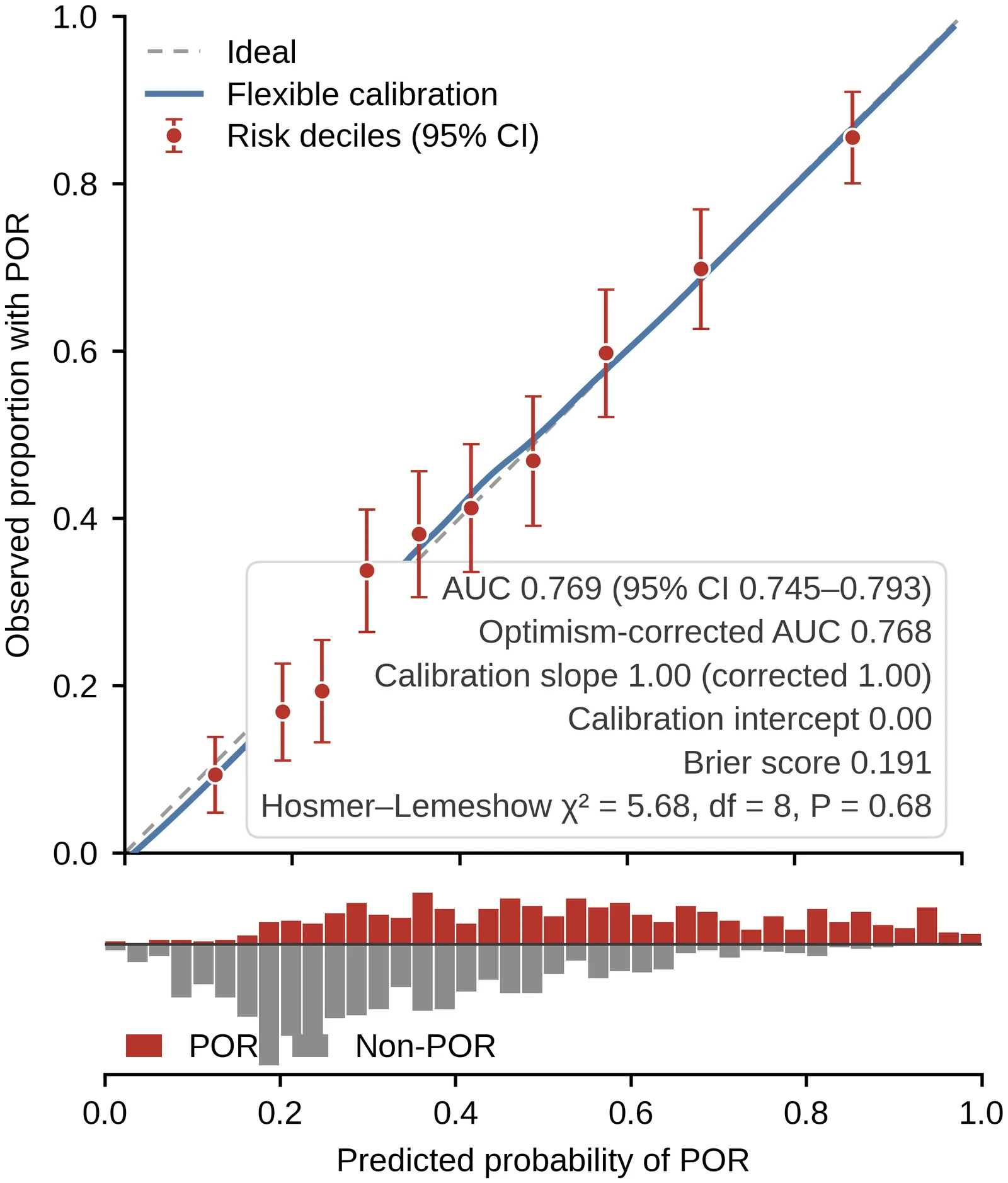
Calibration of the primary two-marker model in the development cohort (n = 1,597). Risk deciles (points, with 95% confidence intervals) and a flexible calibration curve are plotted against the ideal 45° line. The strip below shows the distribution of predicted risks for cycles with (above the axis) and without (below) a poor ovarian response. For the fixed two-marker specification, calibration slope 1.00 (bootstrap-corrected 1.00); calibration intercept 0.00; Brier score 0.191; Hosmer–Lemeshow χ^2^ = 5.68, df = 8, P = 0.68. POR, poor ovarian response.

**Figure 5:**
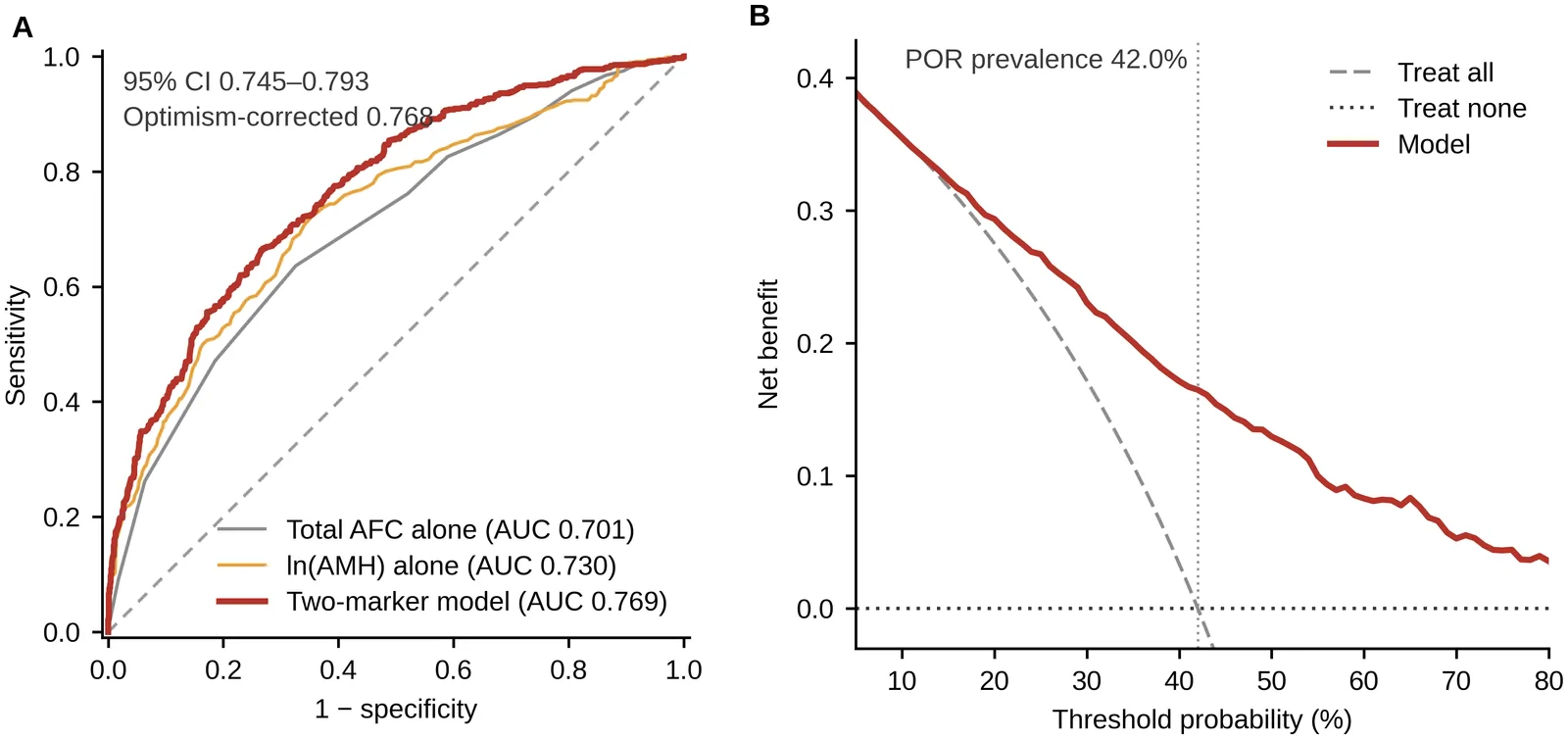
Discrimination and clinical utility of the primary two-marker model (development cohort, n = 1,597). **(A)** ROC curves for the two-marker model (AUC 0.769, 95% CI 0.745–0.793; fixed-specification bootstrap-corrected 0.768) and for each reserve marker alone (ln[AMH] 0.730; total AFC 0.701). The two-marker model discriminates better than either marker alone: ΔAUC +0.039 (95% CI 0.024 to 0.054) against ln(AMH) and +0.068 (0.047 to 0.088) against total AFC, both P <0.001 on DeLong’s test. **(B)** Decision curve analysis: net benefit of the model against treat-all and treat-none strategies across threshold probabilities. The dotted vertical line marks the observed poor-response prevalence of 42.0%. AFC, antral follicle count; AMH, anti-Müllerian hormone; AUC, area under the curve; ROC, receiver operating characteristic.

The extended four-variable model performed almost identically (apparent AUC 0.770, optimism-corrected 0.768; Brier 0.190; Hosmer–Lemeshow P = 0.97), and its predicted risks correlated with the primary model’s at r = 0.99 (**Supplementary Table S4**, **Supplementary Figures S4**-**S6**). A 7:3 split-sample analysis agreed with the bootstrap result (validation AUC 0.772, 95% CI 0.728–0.811; **Supplementary Table S6**); the same split also reproduced AFC ≤5 as the Youden-optimal AFC boundary in both partitions.

With the verified record-level centre labels, POR rates were 40.0% in Lishui (239/597), 44.2% in Wenzhou (208/471), 41.2% in Jinhua (138/335), and 44.3% in Huzhou (86/194); there was no strong evidence of between-centre heterogeneity (χ^2^ = 2.37, df = 3, P = 0.50; **Supplementary Table S8**). Adding centre indicators barely changed the reserve-marker estimates (AFC OR 0.843 before and after adjustment; ln(AMH) OR 0.310 and 0.308). In centre-held-out analysis of the fixed two-marker specification, AUCs ranged from 0.725 to 0.800, calibration intercepts from −0.10 to 0.13, and slopes from 0.81 to 1.14 (**Supplementary Table S8**; **Supplementary Figure S3**). Failed retrieval was less consistent across centres, ranging from 3.2% to 8.2% (χ^2^ = 11.30, df = 3, P = 0.010). The centre analyses therefore support reasonable internal consistency for the primary POR model, but not independent external validation, while the rare failed-retrieval endpoint remains more centre-sensitive.

For the 62 failed retrievals, Firth penalised-likelihood regression showed inverse associations with both total AFC (OR 0.781 per follicle, 95% CI 0.689–0.885) and ln(AMH) (OR 0.523, 95% CI 0.396–0.690), both P <0.001 (**Supplementary Table S3**). The same ordering was visible in the three-tier summary: failed retrieval occurred in 12.0% of cycles with both markers low and 1.1% of those with neither marker low. Because there were only 62 events and the failed-retrieval rate varied across centres, this analysis should remain exploratory.

## 4 Discussion

In the primary cohort of 1,597 cycles, 42.0% met the POR definition. Most of the pretreatment predictive information came from AMH and AFC. Together, the two markers separated lower- and higher-risk parts of this already DOR-restricted cohort. At the selected thresholds, POR rates were 23.9%, 47.1% and 78.8% across the three descriptive tiers. The middle tier fell to 39.5% when the *post hoc* exclusions were removed. The main finding is therefore the risk gradient, not the exact percentage attached to any one tier.

The most informative part of the cross-classification may be the two discordant cells. Their observed POR rates differed by only 3.3 percentage points and their median oocyte yield was the same, but the 95% confidence interval for the rate difference extended from −6.2 to +12.7 percentage points. Equivalence was supported only at the wider 15-point margin, and the similarity disappeared when the AMH cut-off was changed to 0.78 ng/mL. We therefore retain all four cells and present the three-tier collapse as a pragmatic, threshold-specific summary rather than as evidence that isolated low AMH and isolated low AFC are clinically interchangeable.

AMH and AFC reflect overlapping aspects of the recruitable follicle pool, but they also have different sources of measurement error: assay variation for AMH and operator-related variation for AFC. Concordantly low results might therefore be more informative than a single low result, although this mechanism was not tested here. Published work in discordant-marker populations has found that AFC can outperform AMH for oocyte yield (19), which is consistent with our own observation that the two single-marker cells were similar at one AMH threshold but not another. This biological explanation should be read as a hypothesis, not as a result of the present study.

Basal FSH and age added little after adjustment for AMH and AFC. Age carries substantial prognostic weight in unselected populations (24, 25); inside a band restricted to women aged 35 years or younger, and already restricted again to diminished reserve, the two markers appear to carry that information. This is a statement about discrimination within this restricted age and reserve range, not a recommendation to base clinical decisions on one type of marker rather than another. We did not test whether counselling based on AMH/AFC rather than age improves any clinical outcome. Within the extended four-variable robustness model, total AFC departed from strict linearity (P = 0.001). Replacing its linear AFC term with a restricted cubic spline changed overall discrimination, accuracy, and net benefit very little, although some individual probabilities moved substantially (**Supplementary Table S11**). Because this sensitivity analysis retained ln(AMH), basal FSH and age, it should not be read as a direct spline refit of the primary two-marker calculator. The primary presentation retains a linear AFC term for interpretability, so estimates at the extremes of the AFC range should be treated as approximate.

The added clinical information here is modest, but it is different from what POSEIDON or later-stage prediction models provide. POSEIDON identifies a young low-reserve phenotype; it does not attach an absolute pretreatment POR probability to a particular AMH–AFC pattern. Teng et al. (18), the closest AMH/AFC cut-off study in young women, focused on identifying mildly reduced reserve before POSEIDON-defined DOR and on subsequent reproductive outcomes. Our analysis starts within DOR and asks a different, earlier treatment-pathway question: how much POR risk can be estimated before protocol choice or intracycle response is known? Other recent models gain discrimination by adding exactly those later variables (16, 17, 26). The answer from AMH and AFC alone is useful but limited. An AUC of 0.768 is only moderate. Meta-analytic evidence confirms that AMH and AFC predict ovarian response in broader IVF populations (27), but their range is narrower in our DOR-selected cohort. The model can separate broad risk groups, but it is not a precise individual-risk calculator. It may help frame expectations before stimulation; it does not identify a treatment that improves outcome. Evidence for interventions such as dual stimulation or oocyte accumulation remains mixed (28–30), even though cumulative outcomes generally improve with greater oocyte yield (31).

The large nominal events-per-variable ratio does not change that conclusion. With 671 events for two final coefficients, imprecision from a small sample is unlikely to explain the moderate AUC. The more important uncertainty comes from how the model was developed: the final two-marker specification followed a data-informed comparison with the four-variable model, and the tier boundaries were not externally fixed. An EPV of 335 does not reflect either of those choices. The bootstrap also refitted the final specification rather than replaying the earlier LASSO and simplification steps, so it does not capture the whole development process (21, 22). Adding more patients with the same two measurements would probably tighten confidence intervals more than it would improve discrimination. A materially better model will likely need genuinely new information, for example, reproducible longitudinal reserve measures, previous stimulation response where available, or markers related to oocyte competence rather than reserve alone.

Protocol choice is another reason not to treat these probabilities as portable constants. GnRH-antagonist use may be associated with POR because of confounding by indication, but protocol also sits downstream of AMH and AFC in routine care: clinicians often choose a regimen after seeing the reserve markers. If treatment selection then changes oocyte yield, part of the model’s absolute risk estimate reflects prescribing patterns in these four centres during 2022–2025. A centre that uses different protocols, or the same centre after its practice changes, may therefore keep a similar rank ordering but lose calibration. External validation—and recalibration when needed—would be required before using the model in another practice setting.

The three-tier scheme is a descriptive risk-stratification framework, not a gatekeeping instrument. The ASRM committee opinion cautions that ovarian-reserve testing should not be used alone to refuse IVF treatment (6). AMH is more informative about ovarian response than reproductive potential: in IVF it predicts oocyte yield and cycle cancellation more consistently than pregnancy outcomes (32), while in women attempting natural conception, ovarian-reserve biomarkers did not predict reduced fecundability (33). Lower AMH is associated with lower cumulative live birth after IVF, but it does not imply a zero chance of live birth (34). These estimates should therefore sit within broader infertility counselling, not function as a stand-alone treatment rule (35).

Some limitations were built into the study from the outset. The retrospective design leaves treatment choice and unmeasured clinical factors open to confounding, and there is still no independent external cohort. The restored DOR file contains a distinct participant ID for every eligible record: no ID repeats among the 1,754 eligible records or the 1,597 records in the primary cohort. This supports the one-record-per-ID structure reported by the centres, although independent de-identification means that cross-centre duplication under different IDs cannot be excluded with absolute certainty. AMH assays were not harmonised and the dataset does not retain a per-cycle platform identifier, so the AMH thresholds are assay-dependent. Oocyte yield is also an intermediate endpoint; we did not model embryo competence/euploidy or cumulative live birth (9, 36, 37). Finally, the results apply only to the population analysed here, including the BMI range of 18.5–30 kg/m^2^. These are important boundaries for the calculator and for the tier estimates.

Other limitations emerged only once the data were analysed and the revision questions were worked through. The PCOS and AMH ≥5 ng/mL exclusions were *post hoc* and were made while outcomes were accessible; without them, the middle-tier POR rate drops from 47.1% to 39.5%. The AFC ≤5 boundary was informed by POSEIDON’s <5 criterion, is not identical to it and was not registered. The split-sample and in-bag/out-of-bag checks make that boundary more credible internally, but they do not replace external validation. Likewise, the bootstrap and centre-held-out analyses refit the final model specification without replaying the earlier LASSO and model-simplification steps, so their performance estimates remain conditional on that specification (21, 22). The native centre labels have removed the previous reconstruction problem, but all four hospitals are in the same province, AMH assays were not harmonised, and failed retrieval varied across centres. Geographic and measurement transportability therefore remain unresolved. Prospective validation with harmonised assays and clinically relevant downstream outcomes is still needed.

## 5 Conclusion

In young women with DOR, AMH and AFC provided moderate pretreatment discrimination for POR. The two single-marker subgroups looked similar at the clinically selected AMH threshold used in the primary analysis, but equivalence was not established and the finding changed when the AMH cut-off changed. The descriptive three-tier rates were 23.9%, 47.1%, and 78.8%; the middle tier fell to 39.5% when the *post-hoc* phenotype-cleaning exclusions were removed. AFC ≤5 was reproduced in the 7:3 split and was re-selected in 92.0% of bootstrap in-bag samples with out-of-bag evaluation. Centre-held-out AUCs for the fixed two-marker specification ranged from 0.725 to 0.800. Together, these analyses strengthen the internal assessment but do not remove uncertainty from the data-informed development pathway or provide independent external validation. The estimates may help frame pretreatment risk discussions; they are not a treatment rule and should not determine access to care.

## Data Availability

The individual-cycle clinical dataset is not publicly available. Two of the four collaborating centres authorised use of their records for this analysis only and did not consent to public deposition of individual-level clinical data, and we have respected that decision rather than deposit a partial dataset. The de-identified dataset can be provided to the journal by the corresponding author if the editors request it during review or after acceptance. Any wider release would require written approval from each contributing institution and would remain subject to applicable privacy regulations. The analysis code, the data dictionary, the software-environment notes and source-data files containing every value underlying the tables and figures are supplied as Supplementary Material, so the tables and figures can be reproduced without access to the individual-level records, and the full analysis can be rerun once access has been granted.

## Supplementary material

The Supplementary Material for this article can be found online at: https://www.frontiersin.org/articles/10.3389/fendo.2026.1968836/full#supplementary-material. The **Supplementary Material** comprises: **Supplementary Note S1** (extended methods), **Supplementary Tables S1**-**S11** and the legends for **Supplementary Figures S1**-**S6** (Data Sheet 1); **Supplementary Figures S1**-**S6** (image files); the reporting checklists, TRIPOD (2015) and STROBE (Data Sheet 2); the risk calculator implementing the primary two-marker equation and the three-tier rule, with input guards that flag out-of-range inputs and suppress the estimate; and the analysis code, software-environment notes, data dictionary and source-data files sufficient to regenerate the reported tables and figures once data access has been granted.
