# Supplementary material for: Pretreatment AMH–AFC stratification of poor-ovarian-response risk in young women with diminished ovarian reserve: a multicentre retrospective cohort study
Source: Front Endocrinol (Lausanne). 2026 Sep 28;17:1968836. doi: 10.3389/fendo.2026.1968836 (PMC13654850; doi:10.3389/fendo.2026.1968836)
Supplement: Supplementary file 1 [file SupplementaryFile1.docx]

**Reporting checklists — TRIPOD (2015) and STROBE**

Study type: development and internal validation of a multivariable clinical prediction model. There is no external-validation data set, so TRIPOD items that apply only to a separate validation cohort (10c, 12, 19a) are marked N/A; internal validation is reported under item 17.

Locators refer to the accompanying manuscript by section number, table, figure or supplementary item. We have deliberately not used page and line numbers: the manuscript was shortened by about 30% at revision and will be repaginated again at typesetting, so anchors of this kind are the only ones that stay true. Material moved out of the main text during that revision is in Supplementary Note S1 and is cited as such. The Ethics statement also distinguishes the January 2022–December 2025 clinical-record period from the 16 June 2026 approval for retrospective secondary research use, and records the collaborating-centre authorisations covering those historical de-identified records.

**TRIPOD 2015 checklist**

| **Item** | **Checklist item** | **Where it is reported** |
| --- | --- | --- |
| 1 | Title — Identify the study as developing and/or validating a multivariable prediction model, the target population, and the outcome. | Title (development and internal validation; women aged ≤35 years with DOR; poor ovarian response) |
| 2 | Abstract — Structured summary of objectives, design, setting, participants, sample size, predictors, outcome, statistical analysis, results and conclusions. | Abstract |
| 3a | Background — Explain the medical context and rationale for developing the model, including references to existing models. | §1, paragraphs 1–3 (existing nomograms, refs 16, 17, 19) |
| 3b | Objectives — Specify objectives, including whether the study is development, validation, or both. | §1, final sentence (two aims: stratification; development and internal validation) |
| 4a | Source of data — Describe the study design or source of data. | §2.1, paragraph 1 (retrospective multicentre cohort, pooled four-centre registry) |
| 4b | Source of data — Specify key study dates (accrual start and end). | §2.1, paragraph 1 (January 2022 – December 2025) |
| 5a | Setting — Key elements of the setting; number and location of centres. | §2.1, paragraph 1 (four centres, Zhejiang, China); Figure 1; Supplementary Table S8; Supplementary Figure S3; Supplementary Note S1.2 |
| 5b | Eligibility — Describe eligibility criteria for participants. | §2.1, paragraph 2 (DOR definition; BMI, AFC and cycle-length exclusions; PCOS and AMH ≥5 ng/mL); Figure 1; Supplementary Note S1.4 |
| 5c | Treatments received — Details of any treatments received, if relevant. | §2.1, paragraph 1 (IVF/ICSI); §2.2, paragraph 2 and §3.2 (GnRH-antagonist protocol as an adjustment variable); Supplementary Table S7 |
| 6a | Outcome — Define the outcome predicted, including how and when assessed. | §2.1, paragraph 3 (poor ovarian response, ≤3 oocytes; failed retrieval, 0 oocytes) |
| 6b | Outcome — Actions to blind outcome assessment. | N/A — the outcome is an objective laboratory oocyte count taken from the clinical record |
| 7a | Predictors — Define all predictors, including how and when measured. | §2.2 (ten pretreatment candidates, all recorded at the first consultation; AMH assay and AFC measurement) |
| 7b | Predictors — Actions to blind predictor assessment. | N/A — all predictor values were recorded before the outcome occurred |
| 8 | Sample size — Explain how the study size was arrived at. | §2.1 and §3.1 — all eligible cycles in the registry; no a priori size calculation. Events per variable 335.5 for the primary model (Table 2 footnote). See §4, Limitations. |
| 9 | Missing data — How missing data were handled. | §2.3, paragraph 1 (complete-case analysis); Figure 1 (records excluded for missing AFC or cycle length) |
| 10a | Development — How predictors were handled in the analyses. | §2.3, paragraph 1 (ln[AMH]); §2.3, final paragraph and Supplementary Note S1.6 (restricted cubic spline check); Supplementary Table S11 |
| 10b | Development — Type of model, model-building procedures and internal-validation method. | §2.3, paragraphs 2–3 (logistic regression; LASSO with 5-fold cross-validation; bootstrap optimism correction, 1,000 replicates) |
| 10c | Validation — How predictions were calculated in the validation data. | N/A — no separate external-validation data set |
| 10d | Performance — Measures used to assess and compare model performance. | §2.3, paragraph 3 (AUC, flexible and decile calibration, slope and intercept, Brier score, Hosmer–Lemeshow, decision curve analysis, DeLong’s test) |
| 11 | Risk groups — How risk groups were created, if done. | §2.3, paragraph 4 (AFC ≤5 and AMH <0.5 ng/mL; pre-specified 1-degree-of-freedom contrast; TOST equivalence margins); Table 3 |
| 12 | Validation — Differences between development and validation data. | N/A — development and internal validation only |
| 13a | Participants — Flow of participants, numbers with and without the outcome; diagram. | Figure 1; §3.1, paragraph 1 |
| 13b | Participants — Characteristics, including numbers with missing data. | Table 1; §3.1, paragraph 2 |
| 14a | Development — Number of participants and outcome events in each analysis. | §3.1 (1,597 cycles; 671 poor-response events, 42.0%; 62 failed retrievals); Table 2 and Table 3 |
| 14b | Development — Unadjusted association between each candidate predictor and the outcome. | Supplementary Table S1; Supplementary Figure S2A; §3.2, paragraph 1 |
| 15a | Model specification — Full model, all coefficients and the intercept. | Table 2 with its footnote (primary two-marker model: logit[P] = 0.305 − 0.171 × AFC − 1.170 × ln[AMH]); Figure 3 legend; extended four-variable model in Supplementary Table S2 |
| 15b | Model specification — Explain how to use the model. | Figure 3 (nomogram, with a worked example); the risk calculator supplied as Supplementary Material; §3.3 and Table 3 for the three-tier rule |
| 16 | Model performance — Report performance measures, with confidence intervals. | §3.4, paragraph 1 (AUC 0.769, 95% CI 0.745–0.793; optimism-corrected 0.768; Brier 0.191; Hosmer–Lemeshow P = 0.68; ΔAUC on DeLong’s test); Figures 4 and 5; Table 2 footnote |
| 17 | Model updating and internal validation — Results of validation or updating. | §3.4, paragraphs 1–2; Supplementary Table S4 (bootstrap optimism correction); Supplementary Table S6 (7:3 split-sample sensitivity analysis) |
| 18 | Limitations — Discuss limitations. | §4, limitations (retrospective design; distinct participant IDs for all eligible/primary records, with cross-ID duplication not fully excludable after de-identification; verified record-level centre labels but all centres within one regional network; post hoc phenotype-cleaning exclusions; no independent external validation; threshold and assay dependence; complete-case analysis) |
| 19a | Validation — Interpret results with reference to the validation data. | N/A — no external validation |
| 19b | Interpretation — Overall interpretation considering objectives, limitations and similar studies. | §4, paragraphs 1–6; §5 |
| 20 | Implications — Potential clinical use and implications for further research. | §4, paragraphs 5–6 (counselling and stimulation planning; not a gate-keeping instrument); §5 |
| 21 | Supplementary information — Availability of protocol, calculator and data sets. | Supplementary material section; Data availability statement; Supplementary Note S1; the risk calculator, analysis code, data dictionary and source-data files in the Supplementary Material |
| 22 | Funding — Source of funding and the role of the funders. | Funding section — Scientific Research Project of Lishui University, grant X111014; the funder had no role in design, analysis, writing or the decision to submit |

Reference: Collins GS, Reitsma JB, Altman DG, Moons KGM. Transparent Reporting of a multivariable prediction model for Individual Prognosis Or Diagnosis (TRIPOD): the TRIPOD statement. Ann Intern Med. (2015) 162:55–63. doi: 10.7326/M14-0697

**STROBE checklist (cohort studies)**

| **Item** | **Checklist item** | **Where it is reported** |
| --- | --- | --- |
| 1 | Title and abstract — (a) indicate the design in the title or abstract; (b) give a balanced summary. | (a) Title (“multicentre retrospective cohort study”); (b) Abstract |
| 2 | Background and rationale — Scientific background and rationale. | §1, paragraphs 1–2 |
| 3 | Objectives — State specific objectives, including any pre-specified hypotheses. | §1, final sentence |
| 4 | Study design — Present key elements of the design early in the paper. | §2.1, paragraph 1 |
| 5 | Setting — Setting, locations and relevant dates. | §2.1, paragraph 1 (four centres; January 2022 – December 2025); Supplementary Note S1.2 |
| 6 | Participants — Eligibility criteria, sources and methods of selection. | §2.1, paragraph 2; Figure 1; Supplementary Note S1.4 |
| 7 | Variables — Define outcomes, exposures, predictors and potential confounders. | §2.1, paragraph 3 (outcomes); §2.2 (predictors) |
| 8 | Data sources and measurement — Sources and details of assessment for each variable. | §2.2, paragraph 1 (AMH assay platforms, AFC on transvaginal ultrasound, cycle days 2–4) |
| 9 | Bias — Describe efforts to address potential sources of bias. | §2.1, paragraph 2 and Supplementary Note S1.4 (exclusion rationale and full-cohort sensitivity analysis, Supplementary Table S9); §3.2 (confounding by indication for protocol); §4, final paragraph |
| 10 | Study size — Explain how the study size was arrived at. | §2.1 and §3.1 — all eligible cycles |
| 11 | Quantitative variables — How quantitative variables were handled and any groupings. | §2.3, paragraph 1 (ln[AMH]); §2.3, paragraph 4 (AFC and AMH thresholds); Supplementary Table S11 (spline check) |
| 12 | Statistical methods — Methods, subgroups, missing data, sensitivity analyses. | §2.3 in full; Supplementary Note S1.5 and S1.6 |
| 13 | Participants — Numbers at each stage; reasons for exclusion; diagram. | Figure 1; §3.1, paragraph 1 |
| 14 | Descriptive data — Characteristics of participants and missing data. | Table 1; §3.1, paragraph 2 |
| 15 | Outcome data — Report numbers of outcome events or summary measures. | §3.1 (671 poor-response events); §3.3 and Table 3 (by tier); §3.4, final paragraph (62 failed retrievals) |
| 16 | Main results — Adjusted estimates and their precision; category boundaries. | Table 2; §3.2, paragraph 2; Table 3 and §3.3 (tier boundaries and rates with 95% CIs) |
| 17 | Other analyses — Subgroups, interactions and sensitivity analyses. | §3.4, paragraph 3 (centre-stratified); Supplementary Tables S5–S11; Supplementary Note S1.6 |
| 18 | Key results — Summarise key results with reference to the objectives. | §4, paragraphs 1–2; §5 |
| 19 | Limitations — Limitations, with the direction and magnitude of any potential bias. | §4, final paragraph |
| 20 | Interpretation — Cautious overall interpretation in the light of other evidence. | §4, paragraphs 3–6 (comparison with existing nomograms and with the discordant-marker literature) |
| 21 | Generalisability — External validity of the results. | §4, final paragraph (single pooled cohort; external validation required; not applicable to PCOS, AMH ≥5 ng/mL or BMI outside 18.5–30 kg/m²) |
| 22 | Funding — Source of funding and the role of the funders. | Funding section — Scientific Research Project of Lishui University, grant X111014 |

Reference: von Elm E, Altman DG, Egger M, Pocock SJ, Gøtzsche PC, Vandenbroucke JP; STROBE Initiative. The Strengthening the Reporting of Observational Studies in Epidemiology (STROBE) statement: guidelines for reporting observational studies. Lancet. (2007) 370:1453–7. doi: 10.1016/S0140-6736(07)61602-X
