# Supplementary material for: Pretreatment AMH–AFC stratification of poor-ovarian-response risk in young women with diminished ovarian reserve: a multicentre retrospective cohort study
Source: Front Endocrinol (Lausanne). 2026 Sep 28;17:1968836. doi: 10.3389/fendo.2026.1968836 (PMC13654850; doi:10.3389/fendo.2026.1968836)
Supplement: Supplementary file 2 [file Table1.docx]

**Supplementary Tables S1–S11**

All tables refer to the analytic cohort of n = 1,597 cycles (671 poor-response events, 42.0%; 62 failed retrievals, 3.9%) unless stated otherwise.

**Supplementary Note S1. Extended methods.**

This note provides methodological detail that is kept out of the main text for readability. It also identifies which decisions were part of the original screening workflow and which were introduced post hoc during analysis or revision.

**S1.1 Record provenance and the unit of analysis**

Each record in the restored DOR analytic dataset carries a de-identified participant_id and represents one stimulation cycle. All 1,754 eligible records have distinct participant IDs, and all 1,597 records in the primary cohort also have distinct IDs; there are no repeated participant_id values. The contributing centres reported retaining only the first eligible cycle per woman within the study window. The restored IDs therefore support a one-record-per-ID structure, although independently de-identified IDs cannot completely exclude the same woman being assigned different IDs at different centres. The stimulation cycle remains the unit of analysis throughout. We found one cross-centre pair (different IDs, one from Lishui and one from Wenzhou) with identical values on every clinical field available in the analytic file. The pair was retained because the de-identified data cannot determine whether it is a coincidental match or a duplicate, and removing either record leaves the model coefficients, three-tier separation and AUC unchanged to the reported precision.

**S1.2 Centre of origin**

The restored analytic file contains a record-level centre code (L/W/J/H), corresponding to Lishui, Wenzhou, Jinhua and Huzhou. We therefore no longer infer centre from participant-number blocks. Comparing the native labels with the earlier block reconstruction showed that the previous reconstruction had reversed the order of the Huzhou and Jinhua blocks; 448 of 1,754 records would consequently have carried the wrong centre in that reconstruction. All centre-specific analyses in this revision were rerun from the native labels. Pooled analyses are unchanged because centre does not enter the primary model.

For the present retrospective secondary analysis, the lead ethics approval dated 16 June 2026 was obtained after the January 2022–December 2025 routine clinical records had been generated but before study-specific extraction, pooling and analysis. The authors confirmed that each centre’s signed institutional authorisation covers retrospective use of its de-identified records from that 2022–2025 period in this collaborative study. The approval and centre authorisations therefore govern the research use of historical records; they are not presented as prospective approvals in force when the underlying clinical care occurred.

**S1.3 The clinician-assigned DOR flag**

The registry also carried a clinician-assigned DOR flag. This flag agreed with the AMH/AFC criterion in only 53.8% of the analysed records. The explicit AMH/AFC definition, not the clinician’s label, therefore defines the cohort throughout.

S1.4 Eligibility restrictions and the two post hoc phenotype-cleaning exclusions

The BMI range of 18.5–30 kg/m² is different in kind from the two exclusions described below. It was applied as an eligibility criterion at the first screening step, before the DOR cohort was identified, and it uses no information about ovarian response; the step order is given in the deposited screening log. We do not describe the range as pre-specified: the screening log fixes where it sits in the sequence, but we hold no dated record of when the range was first agreed. One consequence is worth stating plainly. The predictive contribution of BMI was assessed only within this restricted range, so the model should not be extrapolated to women whose BMI falls outside it.

Both exclusions are defined on pretreatment characteristics only, a recorded diagnosis and a baseline AMH value, and neither uses the number of oocytes retrieved. Women with a recorded diagnosis of polycystic ovary syndrome (Rotterdam criteria; n = 139) were excluded because polycystic ovaries can satisfy an AFC-based reserve criterion while the follicular pool is not in fact depleted; in this registry those women had a median AMH of 5.86 ng/mL (maximum 25.0) and a poor-response rate of only 9.4%. Women with AMH ≥5 ng/mL in the absence of PCOS (n = 18) were excluded because that combination is not compatible with diminished reserve; these records had a median AMH of 7.64 ng/mL alongside a median AFC of 4, retrieved a median of 19.5 oocytes (maximum 45), and none met the poor-response definition. Together the two criteria removed 157 of 1,754 records (9.0%). The DOR definition itself was not modified.

The PCOS and AMH ≥5 ng/mL exclusions were made during post hoc phenotype cleaning after the outcome data were accessible. Although the criteria themselves use pretreatment variables, they were not outcome-blind. The excluded groups subsequently showed lower POR rates. Repeating the tier analysis in all 1,754 eligible cycles (Supplementary Table S9) preserved the broad ordering, but the middle-tier POR rate fell from 47.1% to 39.5%; that absolute estimate is therefore materially dependent on the exclusions. Refitting the same primary two-marker model in the full eligible cohort gave an AFC OR of 0.846 (95% CI 0.814–0.879), an ln(AMH) OR of 0.275 (0.232–0.326) and an apparent AUC of 0.795, compared with 0.843, 0.310 and 0.769 in the primary analytic cohort.

**S1.5 Pre-specification**

This retrospective study had no registered statistical analysis plan. The candidate predictor set and the AMH 0.5 ng/mL threshold were based on clinical rationale. All ten pretreatment candidates were examined univariably and then entered into LASSO; a GnRH-antagonist-protocol indicator was added as an eleventh LASSO candidate. The univariable results were not used to screen variables out before LASSO. The final two-marker specification followed a data-informed comparison with an extended four-variable model, and the fixed-specification model bootstrap did not repeat the preceding LASSO or simplification step. The AFC ≤5 operational boundary was informed by POSEIDON’s AFC <5 criterion but is not identical to it and was not registered. Because it coincides with the primary-cohort Youden optimum, we added a seed-2024 70:30 split in which the cut-off was selected in the training set and evaluated in the held-out set, and a 1,000-replicate in-bag/out-of-bag bootstrap that re-selected the AFC cut-off within every resample. These analyses strengthen internal support for the boundary but do not make it externally validated. The two phenotype-cleaning exclusions were post hoc.

**S1.6 Post hoc robustness analyses**

These were conducted after the primary analysis. Within the extended four-variable model (total AFC, ln[AMH], basal FSH and age), the functional form of each continuous predictor was examined with restricted cubic splines and a total AFC × ln(AMH) interaction was tested (Supplementary Table S10). The four-predictor model was also compared with the parsimonious two-marker model by the likelihood-ratio test. Because total AFC departed from linearity in the extended model, we then replaced its linear AFC term with a four-knot restricted cubic spline while retaining ln(AMH), basal FSH and age, and compared that spline model with the corresponding linear-AFC extended model (Supplementary Table S11). This was a functional-form sensitivity analysis of the extended model; it was not a direct spline refit of the primary two-marker nomogram or calculator. We also added threshold-stability analyses that re-selected the AFC boundary in training or bootstrap in-bag samples before evaluating it in held-out observations, and centre-level internal–external cross-validation using the verified record-level centre code.

**S1.7 Software**

Analyses were performed in Python 3.12.3 with numpy 2.4.4, pandas 3.0.2, SciPy 1.17.1, scikit-learn 1.8.0, matplotlib 3.10.8 and openpyxl 3.1.5. The unpenalised logistic maximum-likelihood fit, Firth penalised likelihood, Hosmer–Lemeshow decile test, bootstrap procedures and decision-curve net benefit are contained in the analysis scripts. Random seed 2024 is fixed throughout, including the model bootstraps, LASSO cross-validation folds, 7:3 split and threshold-resampling analyses.

**Supplementary Table S1. Univariable logistic regression and discriminative ability of each candidate pretreatment predictor.**

| **Predictor** | **Crude OR (95% CI)** | **P** | **AUC** |
| --- | --- | --- | --- |
| Age (per year) | 1.047 (1.008–1.087) | 0.017 | 0.541 |
| BMI (per kg/m²) | 1.054 (1.017–1.093) | 0.004 | 0.547 |
| Infertility duration (per year) | 1.016 (0.980–1.054) | 0.381 | 0.516 |
| Total AFC (per follicle) | 0.805 (0.775–0.836) | <0.001 | 0.701 |
| ln(AMH) (per 1 unit) | 0.264 (0.220–0.318) | <0.001 | 0.730 |
| Basal FSH (per IU/L) | 1.063 (1.039–1.087) | <0.001 | 0.580 |
| Basal LH (per IU/L) | 1.011 (0.988–1.034) | 0.353 | 0.524 |
| Basal E2 (per pmol/L) | 1.001 (1.000–1.002) | 0.005 | 0.519 |
| FSH/LH ratio (per 1 unit) | 1.014 (0.976–1.054) | 0.470 | 0.547 |
| Menstrual cycle length (per day) | 1.005 (0.994–1.017) | 0.379 | 0.545 |

Each predictor entered a separate univariable logistic regression against poor ovarian response (≤3 oocytes). The AUC is that of the single predictor. AMH is entered as the natural logarithm; see Methods. AFC, antral follicle count; AMH, anti-Müllerian hormone; AUC, area under the receiver operating characteristic curve; BMI, body mass index; CI, confidence interval; E2, oestradiol; FSH, follicle-stimulating hormone; LH, luteinising hormone; OR, odds ratio.

**Supplementary Table S2. Coefficients of the extended four-variable model on the logit scale.**

| **Term** | **β** | **SE** | **OR (95% CI)** | **P** |
| --- | --- | --- | --- | --- |
| Intercept | −1.120 | 0.717 | — | — |
| Total AFC (per 1 follicle) | −0.171 | 0.020 | 0.843 (0.811–0.877) | <0.001 |
| ln(AMH) (per 1-unit increase) | −1.154 | 0.096 | 0.316 (0.261–0.381) | <0.001 |
| Basal FSH (per 1 IU/L) | 0.008 | 0.013 | 1.008 (0.982–1.034) | 0.551 |
| Age (per 1 year) | 0.042 | 0.022 | 1.043 (1.000–1.088) | 0.052 |

Model equation: logit(P) = −1.120 − 0.171 × AFC − 1.154 × ln(AMH) + 0.008 × FSH + 0.042 × age, with AFC in follicles, AMH in ng/mL, FSH in IU/L and age in years. The predicted probability is P = 1 / (1 + exp[−logit(P)]). This equation reproduces the extended-model nomogram (Supplementary Figure S4). It is not the equation behind Figure 3 or the Excel calculator: both implement the primary two-marker model, logit(P) = 0.305 − 0.171 × AFC − 1.170 × ln(AMH), reported in main Table 2. AFC, antral follicle count; AMH, anti-Müllerian hormone; CI, confidence interval; FSH, follicle-stimulating hormone; OR, odds ratio; SE, standard error.

**Supplementary Table S3. Firth penalised-likelihood logistic regression for failed oocyte retrieval (0 oocytes; 62 events).**

| **Predictor** | **OR (95% CI)** | **P** |
| --- | --- | --- |
| Total AFC (per 1 follicle) | 0.781 (0.689–0.885) | <0.001 |
| ln(AMH) (per 1-unit increase) | 0.523 (0.396–0.690) | <0.001 |

Because failed retrieval was uncommon (62/1,597 cycles, 3.9%), Firth penalised-likelihood regression was used with only two predictors to avoid separation and overfitting. These results are exploratory. AFC, antral follicle count; AMH, anti-Müllerian hormone; CI, confidence interval; OR, odds ratio.

Supplementary Table S4. Fixed-specification bootstrap optimism assessment of the extended four-predictor model.

| **Metric** | **Apparent** | **Optimism** | Bootstrap-corrected (fixed specification) |
| --- | --- | --- | --- |
| AUC | 0.770 | 0.002 | 0.768 |
| Calibration slope | 1.000 | 0.010 | 0.990 |
| Calibration intercept | 0.000 | — | — |
| Brier score | 0.190 | — | — |
| Hosmer–Lemeshow χ² (df = 8) | 2.18 | — | — |
| Hosmer–Lemeshow P | 0.975 | — | — |

Apparent AUC 0.770 (95% CI 0.747–0.794). Optimism was estimated from 1,000 bootstrap resamples in which the fixed extended four-variable specification was refitted in each resample and the drop in performance between the resample and original cohort was averaged. The preceding LASSO and choice of this four-variable specification were not repeated inside the bootstrap, so the correction is conditional on the displayed model rather than the full development process. This table concerns the extended specification; the primary two-marker model’s fixed-specification bootstrap metrics are reported in the main text and Figure 4. A 7:3 split-sample analysis of the extended model is shown in Supplementary Table S6. AUC, area under the receiver operating characteristic curve; CI, confidence interval.

**Supplementary Table S5. Sensitivity analysis: influence of the AMH threshold on the three-tier stratification.**

| **AMH threshold (ng/mL)** | **Tier 1 POR % (n)** | **Tier 2 POR % (n)** | **Tier 3 POR % (n)** | **AFC low only % (n)** | **AMH low only % (n)** | **P (single-marker cells differ)** |
| --- | --- | --- | --- | --- | --- | --- |
| 0.5 (clinical threshold) | 23.9 (727) | 47.1 (596) | 78.8 (274) | 46.4 (455) | 49.6 (141) | 0.560 |
| 0.78 (Youden) | 19.9 (537) | 37.7 (602) | 73.6 (458) | 33.2 (271) | 41.4 (331) | 0.048 |
| 1.0 | 17.9 (335) | 33.6 (687) | 66.1 (575) | 30.5 (154) | 34.5 (533) | 0.407 |

The AFC threshold is held at ≤5 throughout. This boundary was informed by POSEIDON’s AFC <5 criterion but is study-specific. It is also the primary-cohort Youden optimum. The additional split-sample and in-bag/out-of-bag analyses in Supplementary Table S6 evaluate its stability with cut-off selection separated from held-out evaluation; they do not constitute external validation. Tier 1, neither marker low; Tier 2, one marker low; Tier 3, both markers low. At AMH 0.5 ng/mL, the two single-marker cells differ by 3.3 percentage points (95% CI −6.2 to +12.7; P = 0.56). TOST supports equivalence within 15 percentage points (P = 0.007) but not within 10 (P = 0.081), so the merge should not be read as established equivalence. At the Youden-derived AMH threshold of 0.78 ng/mL the two single-marker cells differ (P = 0.048), and the three-tier collapse is no longer justified on that criterion. AFC, antral follicle count; AMH, anti-Müllerian hormone; POR, poor ovarian response.

Supplementary Table S6. Split-sample model performance and internal confirmation of the AFC boundary.

| **Metric** | **Training (n = 1,117; 469 events)** | **Validation (n = 480; 202 events)** |
| --- | --- | --- |
| AUC (95% CI) | 0.770 (0.741–0.797) | 0.772 (0.728–0.811) |
| Brier score | 0.190 | 0.191 |
| Calibration slope | 1.000 | 0.986 |
| Calibration intercept | 0.000 | 0.061 |
| Hosmer–Lemeshow χ² (df = 8) | 3.55 | 9.68 |
| Hosmer–Lemeshow P | 0.896 | 0.288 |
| AFC Youden cut-off | ≤5 | ≤5 |
| Tier 1 POR at AFC ≤5 / AMH <0.5 | 25.5% (126/494) | 20.6% (48/233) |
| Tier 2 POR at AFC ≤5 / AMH <0.5 | 44.0% (188/427) | 55.0% (93/169) |
| Tier 3 POR at AFC ≤5 / AMH <0.5 | 79.1% (155/196) | 78.2% (61/78) |

A stratified random 7:3 split (seed 2024) was used as a sensitivity analysis. The model-performance rows reproduce the extended four-variable sensitivity analysis. For the threshold check, the AFC cut-off was selected by Youden’s index in the training partition and then applied unchanged with the clinically chosen AMH <0.5 ng/mL boundary to the held-out validation partition. AFC ≤5 was optimal in both partitions. The validation-tier POR rates were 20.6%, 55.0% and 78.2%. In a separate 1,000-replicate bootstrap, the AFC cut-off was re-selected in each in-bag sample and the resulting rule was evaluated only in the out-of-bag observations: AFC ≤5 was selected in 920/1,000 (92.0%) repetitions and ≤4 in 80/1,000; mean out-of-bag POR rates were 24.4%, 47.3% and 79.2% (2.5th–97.5th percentiles across repetitions: 19.9–29.7%, 41.9–53.0% and 72.8–86.6%). These are internal stability analyses, not external validation. AUC, area under the receiver operating characteristic curve; CI, confidence interval; POR, poor ovarian response.

**Supplementary Table S7. Sensitivity analysis: model additionally adjusted for GnRH-antagonist protocol.**

| **Predictor** | **Adjusted OR (95% CI)** | **P** |
| --- | --- | --- |
| Total AFC (per 1 follicle) | 0.853 (0.820–0.887) | <0.001 |
| ln(AMH) (per 1-unit increase) | 0.323 (0.268–0.390) | <0.001 |
| Basal FSH (per 1 IU/L) | 1.010 (0.984–1.036) | 0.462 |
| Age (per 1 year) | 1.043 (0.999–1.088) | 0.054 |
| GnRH-antagonist protocol (vs. other) | 0.617 (0.475–0.800) | <0.001 |

Protocol is associated with a lower odds of poor response, but protocol choice is made after the reserve markers are known and is therefore subject to confounding by indication: clinicians select an antagonist protocol for women they expect to respond. The estimate is reported for transparency and is not interpretable as a treatment effect. Adding protocol leaves the AFC and ln(AMH) estimates essentially unchanged, and protocol is not fixed at the first consultation, so it is excluded from the nomogram. AFC, antral follicle count; AMH, anti-Müllerian hormone; CI, confidence interval; FSH, follicle-stimulating hormone; GnRH, gonadotrophin-releasing hormone; OR, odds ratio.

Supplementary Table S8. Centre-stratified analyses using verified record-level centre labels.

(A) Cohort, poor-response rate and baseline reserve by centre

| **Centre** | **n** | **POR, %** | **Failed retrieval, %** | **AMH, median** | **Age, mean** |
| --- | --- | --- | --- | --- | --- |
| Lishui (lead) | 597 | 40.0 | 3.4 | 0.79 | 32.0 |
| Wenzhou | 471 | 44.2 | 3.2 | 0.78 | 32.2 |
| Jinhua | 335 | 41.2 | 3.3 | 0.75 | 32.2 |
| Huzhou | 194 | 44.3 | 8.2 | 0.77 | 32.3 |

Using the record-level centre code, observed POR rates ranged from 40.0% to 44.3% (χ² = 2.37, df = 3, P = 0.50). The four centres contributed 597, 471, 335 and 194 analytic cycles from Lishui, Wenzhou, Jinhua and Huzhou, respectively. Failed retrieval was more variable across centres (3.2%–8.2%; χ² = 11.30, df = 3, P = 0.010). Because only 62 failed retrievals occurred overall, this centre difference is reported descriptively and reinforces the exploratory status of that secondary endpoint.

(B) Internal–external cross-validation of the fixed two-marker specification

| **Held-out centre** | **Training n** | **Test n** | **AUC** | **Calib. intercept** | **Calib. slope** |
| --- | --- | --- | --- | --- | --- |
| Lishui | 1000 | 597 | 0.776 (0.738–0.813) | −0.10 | 1.14 |
| Wenzhou | 1126 | 471 | 0.725 (0.678–0.767) | 0.13 | 0.81 |
| Jinhua | 1262 | 335 | 0.793 (0.743–0.843) | −0.08 | 1.06 |
| Huzhou | 1403 | 194 | 0.800 (0.738–0.863) | 0.01 | 1.00 |

The primary two-marker specification was refitted on three centres and evaluated in the fourth, then repeated so that each centre served once as the held-out set. Held-out AUCs ranged from 0.725 to 0.800, calibration intercepts from −0.10 to 0.13 and calibration slopes from 0.81 to 1.14. This is internal–external cross-validation of the fixed model specification; because the earlier LASSO/model-simplification pathway was not repeated inside each cycle, it does not validate the full model-development process. All four centres also belong to the same regional network, so independent external validation remains necessary.

(C) Reserve-marker coefficients with and without centre fixed effects

| **Predictor** | Primary two-marker model, OR | With centre fixed effects, OR |
| --- | --- | --- |
| Total AFC (per follicle) | 0.843 | 0.843 |
| ln(AMH) (per 1 unit) | 0.310 | 0.308 |

Adding verified centre indicators to the primary two-marker model changed the reserve-marker coefficients negligibly: the AFC OR was 0.843 before and 0.843 after centre adjustment, and the ln(AMH) OR was 0.310 and 0.308. A likelihood-ratio test for the three centre indicators gave χ² = 3.34, df = 3, P = 0.34. These results support consistency of the fixed model within the four contributing centres but should not be read as evidence of transportability beyond this network. AFC, antral follicle count; AMH, anti-Müllerian hormone; AUC, area under the curve; CI, confidence interval; OR, odds ratio; POR, poor ovarian response.

Supplementary Table S9. Sensitivity analysis: primary two-marker model and tier estimates in the full eligible cohort without the two post hoc phenotype-cleaning exclusions.

| **Measure** | **Primary analytic cohort (n = 1,597)** | **Full eligible cohort, no phenotype-cleaning exclusions (n = 1,754)** | **Difference** |
| --- | --- | --- | --- |
| Poor-response events, n (%) | 671 (42.0%) | 684 (39.0%) | −3.0 pp |
| Total AFC, adjusted OR (95% CI) | 0.843 (0.811–0.876) | 0.846 (0.814–0.879) | +0.003 |
| ln(AMH), adjusted OR (95% CI) | 0.310 (0.258–0.373) | 0.275 (0.232–0.326) | −0.035 |
| Apparent AUC | 0.769 | 0.795 | +0.026 |
| Tier 1 (neither low) POR %, n | 23.9%, 727 | 23.8%, 752 | −0.1 pp |
| Tier 2 (one low) POR %, n | 47.1%, 596 | 39.5%, 726 | −7.6 pp |
| Tier 3 (both low) POR %, n | 78.8%, 274 | 79.0%, 276 | +0.2 pp |

The primary analysis removes 157 records in two post hoc phenotype-cleaning exclusions (PCOS, n = 139; AMH ≥5 ng/mL without PCOS, n = 18). This sensitivity analysis retains all 1,754 cycles meeting the AMH/AFC DOR definition and refits the same primary two-marker model (total AFC + ln[AMH]) used in Table 2. The two marker coefficients remain similar and apparent AUC is 0.795, but the middle-tier POR rate decreases from 47.1% to 39.5% while the outer tiers change little. The absolute Tier 2 risk is therefore sensitive to these post hoc exclusions and should not be treated as a stable threshold-specific probability until externally validated. AFC, antral follicle count; AMH, anti-Müllerian hormone; AUC, area under the curve; CI, confidence interval; OR, odds ratio; PCOS, polycystic ovary syndrome; POR, poor ovarian response; pp, percentage points.

**Supplementary Table S10. Additional model-robustness analyses (development cohort, n = 1,597).**

| **Analysis** | **Estimate** | **P value** |
| --- | --- | --- |
| Primary two-variable model (total AFC + ln[AMH]) | AUC 0.769; Brier 0.191 | — |
| Extended four-variable model (+ age + basal FSH) | AUC 0.770; Brier 0.190 | — |
| Incremental value of age + basal FSH (likelihood-ratio test, df = 2) | χ² = 4.3 | 0.12 |
| Non-linearity of total AFC (restricted cubic spline, df = 2) | χ² = 15.0 | 0.001 |
| Non-linearity of ln[AMH] (df = 2) | χ² = 2.7 | 0.26 |
| Non-linearity of basal FSH (df = 2) | χ² = 3.9 | 0.14 |
| Non-linearity of age (df = 2) | χ² = 0.8 | 0.66 |
| Total AFC × ln[AMH] interaction (df = 1) | χ² = 2.8 | 0.09 |

All analyses use the development cohort (n = 1,597) and the natural-log transform of AMH. The primary two-variable model — total AFC and ln(AMH), logit(P) = 0.305 − 0.171 × AFC − 1.170 × ln(AMH), the model implemented in the risk calculator (Supplementary Material) — and the extended four-variable model had almost identical discrimination and calibration, and neither age nor basal FSH added information once total AFC and ln[AMH] were included. Among the continuous predictors only total AFC departed from linearity; the categorical AFC ≤5 cut used in the three-tier scheme partly accommodates this departure. No total AFC × ln[AMH] interaction was detected. Restricted cubic splines used four knots (non-linearity tested on 2 degrees of freedom); the interaction was tested with a single product term (1 degree of freedom). AFC, antral follicle count; AMH, anti-Müllerian hormone; AUC, area under the curve; χ², chi-square statistic from the likelihood-ratio test.

Supplementary Table S11. Total AFC modelled as a restricted cubic spline versus a linear term within the extended four-variable model (development cohort, n = 1,597).

| **Metric** | **Extended model: linear AFC** | **Extended model: spline AFC** |
| --- | --- | --- |
| AUC, apparent | 0.770 | 0.772 |
| AUC, fixed-specification bootstrap-corrected (400 bootstraps) | 0.768 | 0.769 |
| Brier score | 0.190 | 0.189 |
| Decision-curve net benefit, threshold 0.3 | 0.234 | 0.235 |
| Decision-curve net benefit, threshold 0.5 | 0.128 | 0.125 |
| Decision-curve net benefit, threshold 0.7 | 0.059 | 0.072 |

Total AFC departed from linearity in the extended four-variable robustness model (Supplementary Table S10, P = 0.001). We therefore replaced the AFC linear term with a four-knot restricted cubic spline (knots at the 5th, 35th, 65th and 95th centiles: 3, 5, 7 and 14 follicles) while retaining ln(AMH), basal FSH and age, and compared this spline model with the corresponding linear-AFC extended model. This is a functional-form sensitivity analysis of the extended model; it is not a direct spline refit of the primary two-marker nomogram or calculator. Discrimination (optimism-corrected AUC 0.769 vs 0.768), overall accuracy (Brier 0.189 vs 0.190) and decision-curve net benefit were essentially identical. Individual predicted probabilities differed by a mean of 3.1 percentage points (95th centile 7.1; maximum 19.5; Pearson r = 0.99), and 57 of 1,597 cycles (3.6%) crossed a 50% decision boundary. The primary nomogram and calculator retain the prespecified linear AFC presentation for simplicity; individual continuous estimates at the extremes of the AFC range should therefore be treated as approximate. AFC, antral follicle count; AUC, area under the curve.

**Supplementary figure legends**

Supplementary Figure S1. LASSO regularisation path and cross-validated predictor selection (development cohort, n = 1,597). (A) Coefficient paths for the ten candidate pretreatment predictors and a GnRH-antagonist-protocol indicator (11 variables) against log(λ); ln(AMH) and total AFC have the largest standardised coefficients at the cross-validated penalty, while the remaining terms are substantially smaller or zero. (B) Cross-validated binomial deviance (mean ± SD over 5 folds). The dashed line marks the λ selected by cross-validation. AFC, antral follicle count; AMH, anti-Müllerian hormone; LASSO, least absolute shrinkage and selection operator.

Supplementary Figure S2. Forest plots of predictors of ovarian response. (A) Univariable odds ratios for poor ovarian response across the ten pretreatment candidates examined in separate univariable models; the four predictors carried into the extended multivariable model are highlighted. (B) Odds ratios from the extended four-variable model (total AFC, ln[AMH], basal FSH and age), and, below the rule, Firth penalised estimates for the secondary outcome of failed retrieval (0 oocytes; 62 events). The ln(AMH) odds ratio shown here (0.316) is the four-variable estimate; the primary two-marker model gives 0.310 (Table 2). Odds ratios are plotted on a logarithmic scale. AFC, antral follicle count; AMH, anti-Müllerian hormone; CI, confidence interval; OR, odds ratio.

Supplementary Figure S3. Centre-stratified analyses using verified record-level centre labels (see Supplementary Table S8). (A) Observed POR rate in Lishui, Wenzhou, Jinhua and Huzhou with Wilson 95% confidence intervals; the dashed line is the pooled rate (42.0%). (B) Internal–external cross-validation of the fixed two-marker specification, showing AUC and bootstrap 95% confidence intervals for each held-out centre; the dashed line is the primary-cohort apparent AUC (0.769). Held-out AUCs ranged from 0.725 to 0.800. These results assess the fixed specification within the four-centre regional dataset and do not substitute for independent external validation. AUC, area under the curve; CI, confidence interval; POR, poor ovarian response.

Supplementary Figure S4. Nomogram for the extended four-variable model (total AFC, ln[AMH], basal FSH and age). Axes and reading are as in Figure 3; total AFC and ln(AMH) dominate the score (67 and 100 points) while basal FSH and age contribute little (4 and 10 points). Model equation: logit(P) = −1.120 − 0.171 × AFC − 1.154 × ln(AMH) + 0.008 × FSH + 0.042 × age. Worked example (AFC 5, AMH 0.40 ng/mL, FSH 9.0 IU/L, age 33 years → 63% risk). AFC, antral follicle count; AMH, anti-Müllerian hormone; FSH, follicle-stimulating hormone.

Supplementary Figure S5. Calibration of the extended four-variable model in the development cohort (n = 1,597), plotted as in Figure 4. Calibration slope 1.00 (bootstrap-corrected 0.99); calibration intercept 0.00; Brier score 0.190; Hosmer–Lemeshow χ² = 2.18, df = 8, P = 0.97. POR, poor ovarian response.

Supplementary Figure S6. Discrimination and clinical utility of the extended four-variable model (development cohort, n = 1,597), plotted as in Figure 5. (A) ROC curves for the extended model (AUC 0.770, 95% CI 0.747–0.794; optimism-corrected 0.768) and each reserve marker alone (ln[AMH] 0.730; total AFC 0.701). The extended model discriminates better than either marker alone: ΔAUC +0.041 (95% CI 0.025 to 0.056) against ln(AMH) and +0.069 (0.048 to 0.090) against total AFC, both P <0.001 on DeLong’s test. (B) Decision curve analysis; the dotted vertical line marks the observed poor-response prevalence of 42.0%. AUC, area under the curve; ROC, receiver operating characteristic.
